# Trends in incidence, mortality, and DALYs of cystic echinococcosis in Central Asia from 1992 to 2021: an age-period-cohort analysis

**DOI:** 10.3389/fpubh.2024.1504481

**Published:** 2025-01-23

**Authors:** Wulan Talafuhan, Kaibinuer Tuoheti, Ye Lixia, Qi Shuang, Mieyier Yeerjiang, Guzalinuer Aizezi, Wei Jingjing, Peierdun Mijiti

**Affiliations:** ^1^Department of Epidemiology and Biostatistics, School of Public Health, Xinjiang Medical University, Urumqi, Xinjiang, China; ^2^Department of Health Policy and Management, School of Public Health, Xinjiang Medical University, Urumqi, Xinjiang, China; ^3^State Key Laboratory of Pathogenesis, Prevention and Treatment of High Incidence Diseases in Central Asia, First Affiliated Hospital of Xinjiang Medical University, Urumqi, Xinjiang, China

**Keywords:** cystic echinococcosis, global burden of disease, Central Asia, incidence rate, mortality rate, disability-adjusted life years, joinpoint regression model, age-period-cohort analysis

## Abstract

**Background:**

Cystic echinococcosis (CE) is widespread globally but imposes a particularly heavy burden in Central Asia. Despite control measures, disease management remains suboptimal in this region. This study analyzed trends in CE incidence, mortality, and disability-adjusted life years (DALYs) from 1992 to 2021 in Central Asia; compared them with global data; and explored variations by gender, age group, and country to identify critical factors in disease control.

**Methods:**

Using data from the Global Burden of Disease Study 2021 (GBD 2021), we analyzed long-term trends in the incidence, mortality, and DALY rates of CE in Central Asia. The joinpoint regression model was employed to calculate the annual percentage change (APC) and average APC (AAPC) to identify shifts in disease trends. Additionally, an age-period-cohort model was used to assess the impact of various age groups, periods, and birth cohorts on the disease burden.

**Results:**

The number of CE cases increased by 52.13% in Central Asia, while deaths decreased by 57.35%; DALYs decreased only slightly by 10.75%. From 1992 to 2021, CE incidence showed an increasing trend until 2010, then rapidly declined until 2015, and then gradually increased thereafter. The highest incidence rates were among middle-aged and older adult populations. Although mortality and DALY rates decreased across all age groups, the decline was less than the global trend. Gender analysis showed that the incidence rate was significantly higher in males than in females.

**Conclusion:**

Although there have been improvements in the CE disease burden in some Central Asian countries, the overall burden remains significant. This study highlights the importance of considering gender, age, and country-specific disease burdens when formulating public health policies. Future research should continue to monitor these trends and explore targeted prevention strategies within diverse socioeconomic contexts, such as integrating regional socioeconomic factors and public health resources.

## 1 Introduction

Cystic echinococcosis (CE) is a chronic zoonotic disease caused by the larval stages of the *Echinococcus granulosus* tapeworm and is recognized by the World Health Organization (WHO) as one of 17 neglected tropical diseases (NTDs) (1). According to the latest estimates, approximately 188,000 new cases of CE are reported globally each year, resulting in approximately 184,000 disability-adjusted life years (DALYs), averaging approximately 0.98 DALYs per case (2). In regions with limited medical resources, the mortality rate can reach 2–4% (3). CE is widespread globally, particularly in regions such as Argentina, Peru, East Africa, Central Asia, and China (4, 5). Notably, the disease burden is particularly severe in Central Asia due to its unique geographical and climatic conditions (6, 7), wherein a significant number of population are exposed to high-risk environments, particularly herders and farmers (8). Understanding the epidemiological characteristics of CE and its trend in high-burden region is critical to formulate effective regional public health policies and contributes to global control of CE (9). However, study on CE burden in Central Asia was scarce.

Despite significant progress in implementing CE prevention and control programs, achieving ambitious targets for the disease in the WHO 2021–2030 roadmap for NTDs poses multiple challenges (10). Estimates of the disease burden due to CE and analysis of its long-term trend may facilitate the progress toward eliminations. Recent studies had analyzed long-term trends in CE incidence, mortality, and DALYs globally and regionally (9, 11). However, these studies analyzed CE trends using the Global Burden of Disease (GBD) 2019 data, which only extends the GBD data to 2019. Recently, the GBD 2021 data was published, which made substantial updates to the GBD 2019 data, including incorporating 19,189 additional high-quality data sources (e.g., disease registries, hospital records, health surveys) and improvements to the DisMod-MR model, enabling better handling of data sparsity issues (12). Furthermore, detailed analysis on incidence, mortality and DALYs of CE in Central Asia overall and specific countries was not given in this study, although the CE burden in this region was the highest globally. Additionally, these studies did not apply alternative analytical approaches, such as joinpoint regression or age-period-cohort (APC) models, which help identify significant trend shifts and reveal complex age, period, and cohort effects, offering a more detailed understanding of CE’s epidemiological patterns. In this study, we used the GBD 2021 data and analyzed the detailed trends of CE in Central Asia over the past 30 years by age, gender, and country, and compared them with the global trends, which were critical for developing more targeted public health strategies in this high-burden region and might contribute to global efforts in controlling CE.

## 2 Materials and methods

### 2.1 Data source

The number of CE cases, deaths and DALYs, as well as the crude and age-standardized rates of incidence, mortality, and DALYs during 1992–2021 were captured based on the GBD 2021 data, which were retrieved from the Global Health Data Exchange (GHDx, http://ghdx.healthdata.org/gbd-results-tool). The GBD 2021 provides a comprehensive evaluation encompassing 371 diseases and injuries, 288 causes of mortality, and 88 risk factors across various age and gender demographics in 204 countries and territories. The GBD data are primarily derived from national disease surveillance systems, life registration systems, cause of death registration reports, and population survey data. The estimates are produced using DisMod-MR 2.1 Bayesian meta-regression tool. Since the GBD data is comprehensively adjusted by IHME prior to publication, no further cleaning or handling of missing data is required. Our research specifically targeted the disease burden in the Central Asian region, encompassing Armenia, Azerbaijan, Georgia, Kazakhstan, Kyrgyzstan, Mongolia, Tajikistan, Turkmenistan, and Uzbekistan. Since all data were sourced from publicly available databases and no direct human subject research was conducted, ethical approval was not required.

### 2.2 Statistical analysis

We assessed the burden of CE in Central Asia and its trends by analyzing age-standardized incidence rate (ASIR), age-standardized mortality rate (ASMR), age-standardized DALY rate (ASDR), and the total number of cases, deaths, and DALYs, stratified by age groups, gender, and countries, from 1992 to 2021. Rates were reported as per 100,000 population, with 95% uncertainty intervals (UIs) (13, 14). To detect the trends of CE rates, we used the joinpoint regression program (version 5.2.0) developed by the National Cancer Institute. We selected a log-linear model, which was appropriate for exponential or Poisson-distributed data. The significance of each identified trend inflection point was validated using the Monte Carlo permutation test to control the false-positive rate. We estimated the annual percentage change (APC) and the average annual percentage change (AAPC) along with their 95% confidence intervals (CIs). Rates were considered to increase if the AAPC or APC was greater than zero (*p* < 0.05) and to decrease if the AAPC or APC was less than zero (*p* < 0.05); otherwise, rates were considered stable. A maximum of six joinpoints was selected, which provided flexibility to detect meaningful inflection points over the 30-year study period while avoiding overfitting. This selection adhered to the basic rules of the joinpoint Program, such as ensuring sufficient data points and appropriate spacing between joinpoints. The final model was determined using the Monte Carlo permutation test to identify the optimal number of joinpoints based on statistical significance (15, 16). Additionally, we applied an Age-Period-Cohort (APC) model to dissect the impact of age, period, and cohort effects on CE trends, and estimated the parameters including rate ratios (RR), local drift, and net drift for different age groups, periods, and cohorts, along with their 95% CIs using the APC Web Tool (Age Period Cohort Analysis Tool, accessed on 23 August 2024) (17–19). The Wald *χ*^2^ test was used to assess the significance of the estimable parameters and functions, with statistical significance set at *p* < 0.05. Statistical analysis was performed using R software (version 4.2.3).

## 3 Results

### 3.1 The trends of CE burden in Central Asia

Overall in Central Asia, the number of CE cases increased by 52.13% in 1992-2021(from 3,365 in 1992 to 5,120 in 2021), the number of deaths decreased by 57.35% (from 27 in 1992 to 12 in 2021), but the number of DALYs only decreased by 10.75% (from 2,359 in 1992 to 2,106 in 2021). This was similar to the global trend, but the percent change in number of DALYs in Central Asia during the study period was much lower than the global level (57.10%) (Table 1). Notably, males showed higher numbers of cases, deaths, and DALYs than females, reflecting gender disparities in Central Asia (Table 1).

**Table 1:** Incidence, mortality, and DALY number for CE globally and in Central Asia for 1992 and 2021, with trends over 1992–2021.

Item	Incidence number (95% UI)			Mortality number (95% UI)			DALYs (95% UI)		
	1992	2021	Percent change [% (95% UI)]	1992	2021	Percent change [% (95% UI)]	1992	2021	Percent change [% (95% UI)]
Global	92,336 (76,320, 114,190)	148,521 (119,838, 183,224)	60.85 (57.02, 60.46)	3,577 (2,848, 4,398)	1,364 (986, 1775)	−61.88 (−65.39, −59.64)	244,913 (197,567, 301,714)	105,072 (78,967, 133,309)	−57.1 (−60.03, −55.82)
Central Asia	3,365 (2,919, 3,856)	5,120 (4,108, 6,268)	52.13 (40.74, 62.57)	27 (17, 38)	12 (7, 17)	−57.35 (−59.77, −56.44)	2,359 (1733, 3,037)	2,106 (1,411, 2,903)	−10.75 (−18.55, −4.41)
Gender									
Male	1761 (1,527, 2027)	2,623 (2,103, 3,272)	48.98 (37.69, 61.45)	15 (8, 24)	7 (3, 11)	−54.53 (−63.78, −53.77)	1,330 (834, 1805)	1,132 (735, 1,560)	−14.88 (−11.84, −13.55)
Female	1,604 (1,394, 1832)	2,496 (2004, 3,035)	55.59 (43.82, 65.67)	12 (6, 19)	5 (2, 8)	−60.88 (−65.14, −60.88)	1,029 (711, 1,403)	974 (641, 1,348)	−5.42 (−9.82, −3.88)
Countries									
Armenia	273 (219, 339)	292 (233, 354)	7.03 (6.28, 4.55)	1 (0, 1)	0 (0, 0)	−67.6 (−67.66, −66.06)	107 (71, 150)	94 (58, 136)	−12.22 (−17.62, −9.02)
Azerbaijan	239 (189, 297)	364 (285, 444)	52.2 (50.61, 49.44)	2 (1, 2)	1 (0, 1)	−64.05 (−62.61, −61.56)	157 (106, 211)	144 (93, 207)	−8.23 (−12.27, −2.13)
Georgia	285 (225, 344)	216 (182, 256)	−23.96 (−18.82, −25.57)	1 (1, 1)	0 (0, 0)	−72.64 (−73.98, −72.09)	127 (86, 176)	76 (49, 111)	−40.19 (−42.51, −36.91)
Kazakhstan	381 (348, 413)	691 (540, 848)	81.41 (55.24, 105.45)	4 (2, 6)	1 (1, 2)	−69.21 (−71.95, −67.95)	287 (207, 375)	266 (171, 379)	−7.41 (−17.11, 1.08)
Kyrgyzstan	369 (338, 397)	651 (518, 821)	76.29 (53.09, 106.57)	3 (2, 5)	1 (1, 2)	−61.65 (−62.3, −60.47)	247 (175, 327)	249 (166, 349)	0.98 (−5.15, 6.55)
Mongolia	68 (53, 87)	103 (81, 129)	51.97 (54.25, 48.77)	1 (0, 1)	0 (0, 0)	−65.58 (−66.18, −67.03)	72 (38, 118)	47 (30, 65)	−34.94 (−20.62, −44.84)
Tajikistan	468 (404, 555)	816 (648, 1,013)	74.13 (60.7, 82.48)	4 (2, 6)	2 (1, 3)	−56.16 (−55.64, −54.38)	379 (244, 527)	347 (226, 500)	−8.63 (−7.42, −5.12)
Turkmenistan	405 (323, 491)	438 (348, 539)	8.14 (7.68, 9.86)	1 (0, 1)	0 (0, 1)	−43.75 (−47.03, −39.98)	157 (104, 218)	151 (93, 220)	−3.55 (−10.33, 0.56)
Uzbekistan	877 (774, 993)	1,549 (1,249, 1933)	76.53 (61.34, 94.63)	11 (7, 15)	6 (3, 8)	−49.74 (−56.06, −46.59)	827 (603, 1,073)	733 (487, 1,016)	−11.42 (−19.24, −5.31)

UI, uncertainty interval.

The trends in the number of CE cases, deaths and DALYs varied across different countries in Central Asia (Table 1). Kazakhstan experienced the largest increase in the number of CE cases, with a percent change of 81.41%, while Armenia had the smallest increase at 7.03% during the study period. The number of deaths generally decreased in all Central Asian countries, with the highest decrease observed in Georgia (−72.6%). The number of DALYs declined in all countries except Kyrgyzstan, with Georgia and Mongolia showing the most substantial reductions (−40.19% and − 34.94%, respectively). Detailed gender differences in both global and Central Asian countries are provided in Supplementary Table S1.

**Figure 1:**
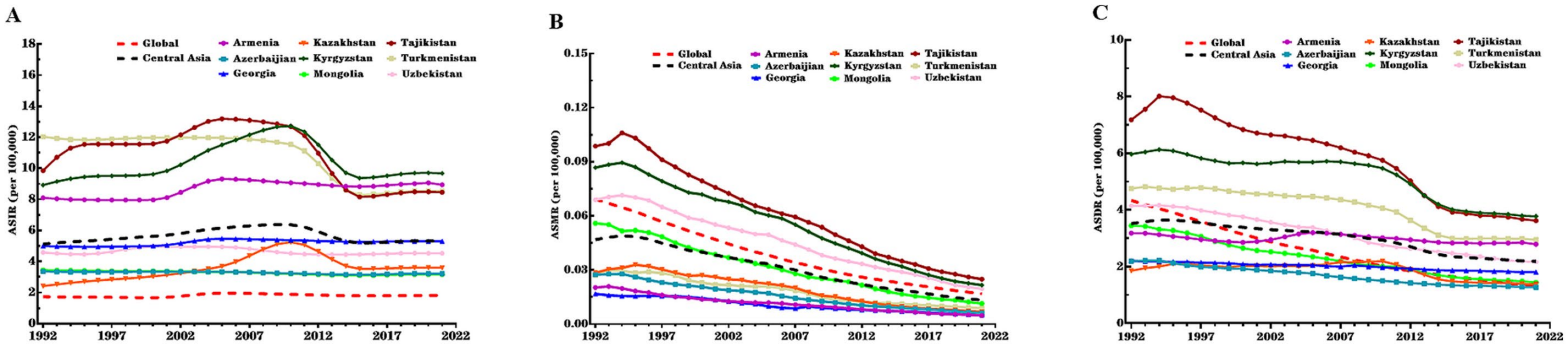
ASIR, ASMR, and ASDR of CE in nine Central Asian countries, Central Asia region overall, and globally, 1992–2021. **(A)** ASIR; **(B)** ASMR; **(C)** ASDR. CE, cystic echinococcosis; ASIR, age-standardized incidence rate; ASMR, age-standardized mortality rate; DALY, disability-adjusted life year; ASDR, age-standardized DALY rate.

Tables 2, 3 and Figure 1 illustrate trends in ASIR, ASMR, and ASDR of CE in Central Asia from 1992 to 2021. The ASIR in Central Asia overall slightly increased from 5.13 per 100,000 population in 1992 to 5.32 per 100,000 population in 2021, with AAPC of 0.15% (95% CI: 0.07, 0.23%). The ASMR and ASDR declined during the study period, with AAPC of −4.24% (95% CI: −4.53, −3.95%) and − 1.66% (95% CI: −1.76,-1.56%), respectively. The joinpoint analysis showed the ASIR in Central Asia progressively increased from 1992 to 2010, with the highest growth observed between 2001 and 2005 (APC = 2.08, 95% CI: 1.75, 2.41%). It subsequently declined sharply from 2010 to 2015 with an APC of −4.28% (95% CI: −4.54, −4.02%), followed by a gradual increase from 2015 to 2021 again (APC = 0.63, 95% CI: 0.45, 0.80%). The ASMR in Central Asia showed a sustained decline from 1992 to 2021, with a brief increase between 1992 and 1994(APC = 3.29, 95% CI: 0.92, 5.71%). Similarly, the ASDR declined overall, with the largest decline observed between 2011 and 2014(APC = -5.49, 95% CI: −6.16, −4.81%), following a brief increase from 1992 to 1995(APC = 1.28, 95% CI: 0.95, 1.61%).

**Table 2:** Age-standardized incidence, mortality, and DALY rates for CE globally and in Central Asia for 1992 and 2021, with trends over 1992–2021.

Item	ASIR, per 100,000 (95% UI)			ASMR, per 100,000 (95% UI)			ASDR, per 100,000 (95% UI)		
	1992	2021	AAPC (%, 95 CI)	1992	2021	AAPC (%, 95 CI)	1992	2021	AAPC (%, 95 CI)
Global	1.73 (1.45, 2.11)	1.82 (1.48, 2.26)	0.17 (0.12, 0.21)	0.07 (0.05, 0.09)	0.02 (0.01, 0.02)	−4.76 (−4.84, −4.68)	4.34 (3.53, 5.32)	1.32 (0.99, 1.69)	−4.03 (−3.97, −4.09)
Central Asia	5.13 (4.47, 5.78)	5.32 (4.31, 6.42)	0.15 (0.07, 0.23)	0.05 (0.03, 0.07)	0.01 (0.01, 0.02)	−4.24 (−4.53, −3.95)	3.52 (2.59, 4.42)	2.19 (1.49, 3.02)	−1.66 (−1.76, −1.56)
Gender									
Male	5.53 (4.83, 6.21)	5.53 (4.48, 6.77)	0.02 (−0.22, 0.27)	0.06 (0.03, 0.09)	0.02 (0.01, 0.03)	−4.16 (−4.33, −4)	4.1 (2.63, 5.45)	2.4 (1.58, 3.26)	−1.83 (−2.08, −1.58)
Female	4.76 (4.15, 5.38)	5.09 (4.13, 6.13)	0.25 (0.19, 0.32)	0.04 (0.02, 0.06)	0.01 (0, 0.02)	−4.42 (−4.71, −4.13)	2.98 (2.14, 3.95)	1.98 (1.32, 2.74)	−1.43 (−1.51, −1.36)
Countries									
Armenia	8.1 (6.56, 9.86)	8.94 (7.21, 10.99)	0.39 (0.31, 0.48)	0.02 (0.01, 0.03)	0 (0, 0.01)	−5.02 (−5.33, −4.71)	3.18 (2.16, 4.45)	2.79 (1.72, 4.08)	−0.45 (−0.55, −0.34)
Azerbaijan	3.36 (2.67, 4.03)	3.22 (2.57, 3.89)	−0.15 (−0.2, −0.11)	0.03 (0.02, 0.04)	0.01 (0, 0.01)	−5.02 (−5.32, −4.73)	2.2 (1.52, 2.94)	1.27 (0.83, 1.81)	−1.87 (−2.05, −1.69)
Georgia	5 (3.97, 6.04)	5.3 (4.43, 6.24)	0.21 (0.15, 0.27)	0.02 (0.01, 0.02)	0.01 (0, 0.01)	−3.88 (−4.53, −3.22)	2.21 (1.5, 3.05)	1.81 (1.19, 2.66)	−0.68 (−0.8, −0.57)
Kazakhstan	2.42 (2.22, 2.61)	3.6 (2.84, 4.37)	1.35 (1.03, 1.67)	0.03 (0.02, 0.04)	0.01 (0, 0.01)	−4.85 (−5.23, −4.47)	1.86 (1.33, 2.41)	1.38 (0.88, 1.96)	−1.01 (−1.29, −0.74)
Kyrgyzstan	8.93 (8.21, 9.54)	9.68 (7.76, 12.06)	0.31 (0.19, 0.43)	0.09 (0.05, 0.13)	0.02 (0.01, 0.03)	−4.68 (−4.98, −4.38)	5.97 (4.25, 7.8)	3.78 (2.54, 5.36)	−1.57 (−1.74, −1.4)
Mongolia	3.45 (2.77, 4.23)	3.16 (2.51, 3.85)	−0.3 (−0.33, −0.28)	0.06 (0.03, 0.09)	0.01 (0.01, 0.02)	−5.32 (−5.91, −4.72)	3.45 (2.12, 5.07)	1.45 (0.95, 2.01)	−2.96 (−3.24, −2.68)
Tajikistan	9.86 (8.47, 11.54)	8.46 (6.85, 10.25)	−0.55 (−0.73, −0.37)	0.1 (0.06, 0.14)	0.02 (0.01, 0.04)	−4.63 (−4.88, −4.39)	7.18 (5.06, 9.56)	3.63 (2.43, 5.1)	−2.33 (−2.48, −2.19)
Turkmenistan	12.04 (9.73, 14.31)	8.54 (6.79, 10.41)	−1.15 (−1.32, −0.98)	0.03 (0.02, 0.04)	0.01 (0.01, 0.01)	−3.84 (−4.31, −3.37)	4.75 (3.17, 6.6)	2.95 (1.83, 4.27)	−1.65 (−1.86, −1.44)
Uzbekistan	4.58 (4.08, 5.1)	4.53 (3.67, 5.57)	−0.02 (−0.08, 0.03)	0.07 (0.04, 0.09)	0.02 (0.01, 0.03)	−4.35 (−4.7, −3.99)	4.14 (3.04, 5.28)	2.17 (1.46, 2.97)	−2.24 (−2.38, −2.11)

ASIR, age-standardized incidence rate; ASMR, age-standardized mortality rate; ASDR, age-standardized DALY rate; CI, confidence interval; UI, uncertainty interval; AAPC, average annual percent change presented for full period.

**Table 3:** Trends in age-standardized incidence, mortality, and DALY rates of cystic echinococcosis in Central Asia and globally from 1992 to 2021, analyzed using joinpoint regression.

Factors	Trend 1		Trend 2		Trend 3		Trend 4		Trend 5		Trend 6	
	Year	APC (95% CI)	Year	APC (95% CI)	Year	APC (95% CI)	Year	APC (95% CI)	Year	APC (95% CI)	Year	APC (95% CI)
Global												
ASIR	1992–2000	−0.55* (−0.61, −0.48)	2000–2005	3.63* (3.44, 3.82)	2005–2015	−1.04* (−1.1, −0.99)	2015–2021	0.31* (0.2, 0.42)				
ASMR	1992–1995	−3.41* (−3.67, −3.15)	1995–2000	−4.57* (−4.74, −4.4)	2000–2008	−5.11* (−5.19, −5.03)	2008–2011	−5.56* (−6.13, −4.99)	2011–2016	−4.52* (−4.72, −4.32)	2016–2021	−4.95* (−5.1, −4.8)
ASDR	1992–1995	−3.32* (−3.57, −3.07)	1995–2002	−4.32* (−4.41, −4.23)	2002–2005	−3.62* (−4.13, −3.1)	2005–2012	−4.65* (−4.74, −4.56)	2012–2021	−3.69* (−3.75, −3.64)		
Central Asia												
ASIR	1992–2001	1.15* (1.09, 1.21)	2001–2005	2.08* (1.75, 2.41)	2005–2010	0.79* (0.58, 1)	2010–2015	−4.28* (−4.54, −4.02)	2015–2021	0.63* (0.45, 0.8)		
ASMR	1992–1994	3.29* (0.92, 5.71)	1994–2006	−3.64* (−3.8, −3.49)	2006–2009	−6.56* (−8.94, −4.11)	2009–2021	−5.45* (−5.59, −5.3)				
ASDR	1992–1995	1.28* (0.95, 1.61)	1995–1999	−1.76* (−2.09, −1.43)	1999–2007	−1.02* (−1.1, −0.93)	2007–2011	−2.45* (−2.79, −2.11)	2011–2014	−5.49* (−6.16, −4.81)	2014–2021	−1.47* (−1.57, −1.36)

APC, annual percent change; ^*^P for trend < 0.05; ASIR, age-standardized incidence rate; ASMR, age-standardized mortality rate; ASDR, age-standardized DALY rate; CI, confidence interval.

Overall, Central Asia had higher ASIR and ASDR and a lower ASMR compared to the global level in all years during the study period. The increase in ASIR during the study period in Central Asia was similar to the global level, while the decrease in ASMR and ASDR was much lower in Central Asia than globally (Table 2). In terms of gender differences, Central Asia had a higher ASIR among males than females, which contrasted with the global pattern where females had a higher ASIR. However, the AAPCs of ASIR among females was higher than males in both Central Asia and globally (Table 2 and Supplementary Table S2).

There were notable variations in ASIR, ASMR, and ASDR of CE across Central Asian countries. In 2021, Kyrgyzstan recorded the highest ASIR (9.68 per 100,000 population) and ASDR (3.78 per 100,000 population), while Mongolia had the lowest ASIR (3.16 per 100,000 population) and Azerbaijan had the lowest ASDR (1.27 per 100,000 population). Kazakhstan showed the largest increase in ASIR during the study period (AAPC = 1.35, 95% CI: 1.03, 1.67%), whereas Turkmenistan exhibited the steepest decrease in ASIR (AAPC = −1.15, 95% CI: −1.32, −0.98%) and the slowest reduction in ASMR (AAPC = -3.84%, 95% CI: −4.31, −3.37) (Supplementary Table S3). In contrast, Mongolia exhibited the fastest declines in ASMR (AAPC = -5.32, 95% CI: −5.91, −4.72) and ASDR (AAPC = −2.96%, 95% CI: −3.24, −2.68). Armenia had the slowest decline in ASDR (AAPC = −0.45%, 95% CI: −0.55, −0.34). Specifically, from 1992 to 2010, Kazakhstan and Kyrgyzstan showed increasing trends in ASIR, followed by a significant decline from 2010 to 2015. Kyrgyzstan and Tajikistan experienced a sharp rise in CE incidence between 2000 and 2004, with a reduction during 2011–2014 (Figure 1 and Supplementary Tables S2, S3).

### 3.2 Net drift and local drift in different age groups

**Figure 2:**
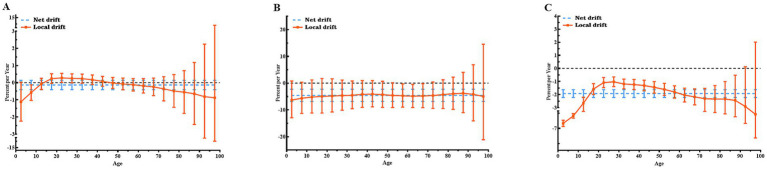
Local and net drift values for incidence, mortality, and DALY rates of CE in Central Asia, 1992–2021. **(A)** Incidence rate; **(B)** Mortality rate; **(C)** DALY rate. Note: Net drift (dotted line) represents the overall annual percentage change across all age groups during the study period. Local drift (continuous line) represents the annual percentage change specific to each age group. A trend is considered statistically significant if its 95% confidence interval (CI) does not include 0. DALY, disability-adjusted life year; CE, cystic echinococcosis.

Figure 2 illustrates the Net drifts and Local drifts values for CE incidence, mortality, and DALYs in Central Asia. Over the study period, the Net drifts for CE mortality and DALYs demonstrated statistically significant reductions of −4.57% (95% CI: −6.82, −2.27%) and − 1.92% (95% CI: −2.22, −1.62%), reflecting substantial reductions in CE mortality and DALYs across the study period, respectively, while the Net drift for CE incidence was not statistically significant.

Local drift values for CE incidence and mortality were largely non-significant across age groups, as their confidence intervals included 0. Exceptions were observed for CE incidence in the 20–24 years age group, which showed an annual increase of 0.28% (95% CI: 0.02, 0.54%), and the 5–9 years age group, which exhibited an annual decrease of −0.58% (95% CI: −1.03, −0.13%). For CE mortality, statistically significant declines were concentrated in the 55–69 years age range, with the greatest reduction occurring in the 65–69 years group (−4.78, 95% CI: −9.18, −0.17%). By contrast, Local drift values for CE DALY rates were predominantly below 0 across most age groups, reflecting overall reductions. The greatest reduction was observed in children under 5 years of age, with an annual decline of −5.85% (95% CI: −6.56, −5.13%). The Net drift and Local drift values for CE incidence, mortality, and DALYs in each specific countries in Central Asia and globally are shown in Supplementary Figures S1–S4.

### 3.3 Age-period-cohort effects on incidence, mortality, and DALY rates of CE

**Figure 3:**
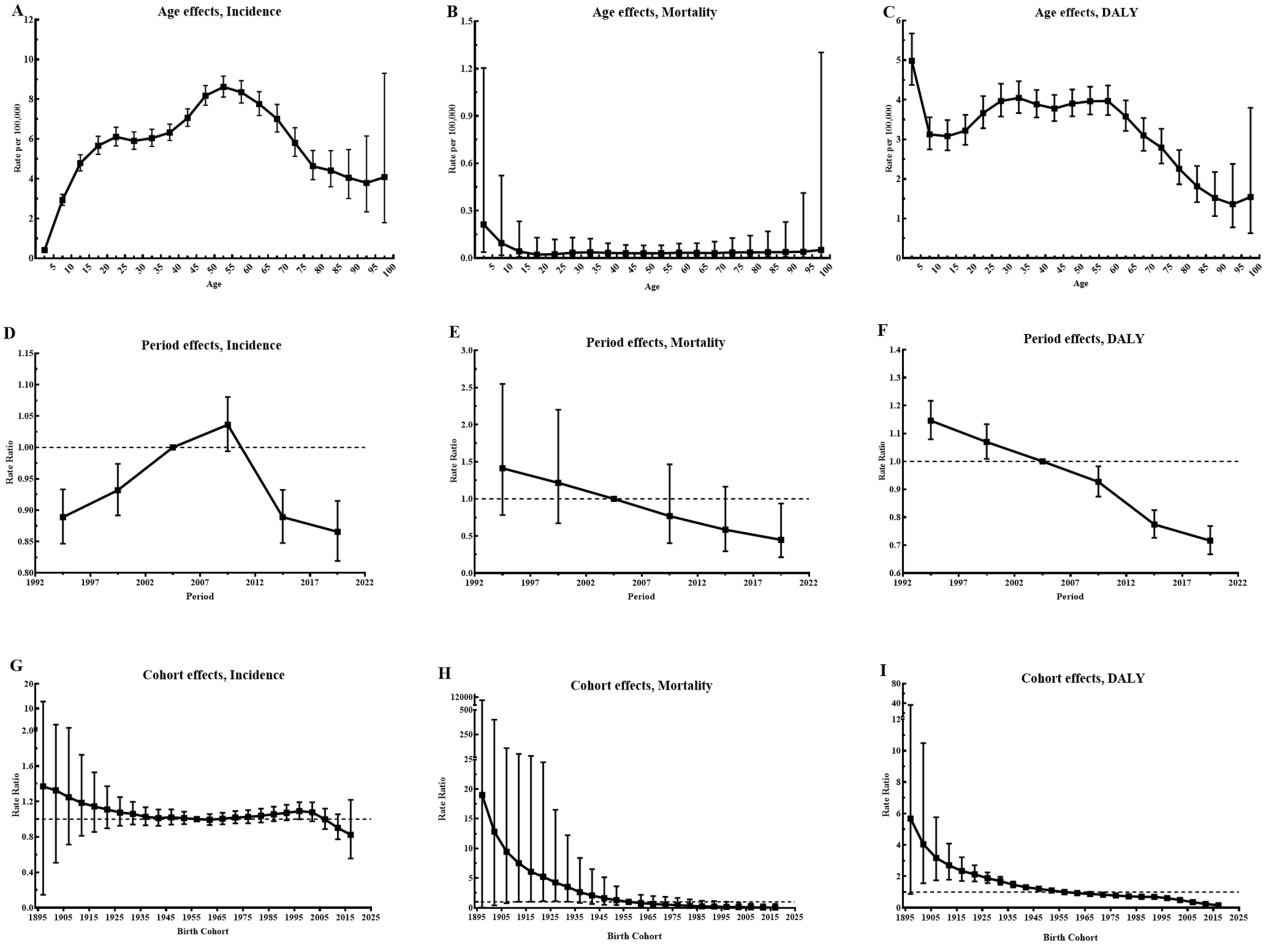
Parameter estimates of age, period, and cohort effects on incidence, mortality, and DALY rates of CE in Central Asia, 1992–2021. **(A)** Longitudinal age curves of CE incidence with corresponding 95% CI; **(B)** Longitudinal age curves of CE mortality with corresponding 95% CI; **(C)** Longitudinal age curves of CE DALY with corresponding 95% CI; **(D)** RR of each period compared with the reference (2002–2006), adjusted for age and nonlinear cohort effects, for incidence, with corresponding 95% CI; **(E)** RR of each period compared with the reference (2002–2006), adjusted for age and nonlinear cohort effects, for mortality, with corresponding 95% CI; **(F)** RR of each period compared with the reference (2002–2006), adjusted for age and nonlinear cohort effects, for DALY, with corresponding 95% CI; **(G)** RR of each cohort compared with the reference (cohort 1955–1959), adjusted for age and nonlinear period effects, for incidence, with corresponding 95% CI; **(H)** RR of each cohort compared with the reference (cohort 1955–1959), adjusted for age and nonlinear period effects, for mortality, with corresponding 95% CI; **(I)** RR of each cohort compared with the reference (cohort 1955–1959), adjusted for age and nonlinear period effects, for DALY, with corresponding 95% CI; Abbreviations: DALY, disability-adjusted life year; CE, cystic echinococcosis; CI, confidence interval; RR, relative risk.

Figure 3 illustrates the estimated age, period, and cohort effects for CE incidence, mortality, and DALYs in Central Asia. According to the longitudinal age curves, the peak incidence rates of CE were observed in children aged 10–14 years and adults aged 50–54 years. By contrast, the age effect on CE mortality was not statistically significant. For DALYs, the rate declined sharply in individuals under 20 years, stabilized with slight fluctuations between 20 and 60 years, and gradually decreased thereafter. The period relative risk (RR) for CE incidence in Central Asia increased during the first two decades but declined in the last decade, reflecting potential improvements in disease control measures. Similarly, period risks for mortality and DALY rates declined continuously, indicating reductions over time. In terms of cohort effects, incidence and mortality risks remained largely stable, while the cohort risk for DALY rates showed a significant decline, indicating a favorable trend. In addition, differences in age, period and cohort effects for incidence were observed across Central Asian countries, as shown in Supplementary Table S4. Notably, relative period risks improved in Kazakhstan, Kyrgyzstan, Tajikistan, and Turkmenistan in recent years, reflecting recent advancements in public health measures. However, younger cohorts in Kazakhstan and Kyrgyzstan exhibited increasing risks, suggesting potential challenges in addressing CE transmission in these populations. Detailed estimates of age, period, and cohort effects for CE incidence, mortality, and DALYs across Central Asian countries and globally are presented in Supplementary Figures S5–S13 and Supplementary Tables S4–S6.

## 4 Discussion

To our knowledge, this is the first study to use the latest GBD 2021 data to conduct a thorough analysis of CE trends in the past 30 years in Central Asia. We found both the number of CE cases and ASIR in Central Asia increased in 1992–2021, while the number of CE deaths and DALYs, ASMR and ASDR decreased, which was similar to the CE trends globally. However, the reductions in mortality and DALYs in Central Asia were substantially slower compared to global levels, highlighting the region’s persistent burden of CE. These findings highlighted the unique challenges faced by Central Asia, which included limited healthcare access, socioeconomic disparities, climate change and traditional livestock practices (6–8, 20, 21).

From 1992 to 2010, CE incidence in Central Asia increased consistently, with the most rapid growth observed between 2001 and 2005. This rise could be attributed to a substantial restructuring of the livestock industry, the unregulated slaughtering of animals, inadequate veterinary control of slaughtering, lack of veterinary public health and increasing number of dog population in both rural and urban areas after 1990s (7, 8). In contrast, between 2010 and 2015, CE incidence significantly declined, reflecting enhanced global and regional public health initiatives (22). In 2009, the WHO Informal Working Group on Echinococcosis (WHO-IWGE) published a consensus on the diagnosis and treatment of cystic and alveolar echinococcosis, providing updated guidelines for diagnosis and treatment (23). The FAO-OIE-WHO Tripartite Agreement, initiated in 2010, fostered collaborations to address zoonotic diseases, including echinococcosis, and may have indirectly contributed to improved disease control efforts in Central Asia (24). However, a moderate rebound in incidence from 2015 to 2021 suggests that the previously observed gains may not have been fully sustained. This trend may reflect persisting gaps in healthcare infrastructure and inconsistent educational campaigns. At the same time, it could also be partially attributed to improved diagnostic capabilities and increased awareness of the disease among the local population and medical community (25). Additionally, the recent expansion of livestock commerce driven by economic initiatives could have contributed to the gradual rise in incidence (26). These findings underscore the need for sustained, long-term interventions to mitigate these challenges effectively.

Our analysis also revealed significant gender and age-specific disparities in CE trends. Males exhibited higher ASIR and ASDR compared to females, contrasting with the global pattern (11). This disparity is likely due to men’s engagement in high-risk occupations such as herding, animal husbandry, and slaughtering, which involve close contact with livestock and dogs, including practices like feeding infected livestock to dogs and working in slaughterhouses with inadequate safety protocols (27–29). Such occupational exposures have been identified as key risk factors in other zoonotic diseases (28). The socio-cultural context in Central Asia, where men predominantly participate in livestock-related activities, may further amplify their risk of exposure (28). Incidence rates exhibited a dual peak among children (10–14 years) and middle-aged adults (50–54 years), reflecting distinct exposure and susceptibility profiles. Children are primarily exposed through environmental contamination, such as contact with Echinococcus eggs in soil near infected dogs, especially in rural areas with inadequate sanitation (25, 30). For middle-aged adults, occupational exposure in high-risk industries such as livestock farming and slaughterhouses remains a significant driver of infection (27, 28). Reductions in DALYs among younger populations, particularly children under 5 years, could indicate the benefits of public health interventions and improved healthcare access.

Significant disparities in CE burden trends were observed across Central Asian countries. For instance, Kazakhstan experienced the largest increase in ASIR, while Mongolia demonstrated the most substantial declines in DALY rates. These disparities likely result from variations in public health infrastructure, policy implementation, and socioeconomic development, which influence healthcare accessibility and disease management (7, 31, 32). Adopting successful strategies from low-burden countries, such as modernized livestock practices and improved meat inspection, could help reduce the CE burden in high-incidence regions. For example, pilot programs in China have demonstrated the effectiveness of enhanced meat inspection and waste management in significantly reducing CE incidence in targeted rural areas. Similarly, collaborative initiatives in Central Asia that draw on livestock management policies from Mongolia have shown promise in mitigating the upward trend of infection rates (29). Regional collaboration and resource-sharing initiatives remain critical for fostering sustainable and effective interventions.

This study is the first to use GBD 2021 data to analyze CE trends in Central Asia, providing a comprehensive analysis of long-term trends and offering critical insights for developing effective disease prevention and control strategies in this high-burden region. Compared to previous studies, this research incorporates several notable advancements. First, it leverages the updated GBD 2021 database, which features a broader range of data sources and a comprehensive update of historical data across all years (14, 33). Additionally, the use of a more advanced DisMod-MR model ensures greater consistency and reliability in estimates, further strengthening the accuracy of trend analysis (14). Second, while earlier studies predominantly focused on global or macro-regional trends, this study provides a detailed examination of Central Asia, stratifying trends by age, gender, and country, and uncovering unique epidemiological characteristics in the region. Finally, the application of Joinpoint and APC models offers a novel analytical perspective, enabling the identification of significant trend shifts and the assessment of age-period-cohort effects. These methodological approaches allow policymakers to prioritize high-risk groups, pinpoint key intervention periods, and develop actionable, targeted public health strategies to address the burden of CE.

Our study has some limitations. First, while the GBD database offers extensive, peer-reviewed data, underreporting and incomplete data, particularly in remote areas with limited healthcare infrastructure, may underestimate the actual burden of CE. In rural and economically disadvantaged regions of Central Asia, limited surveillance systems and healthcare access further exacerbate this challenge, emphasizing the need for more robust data collection (8). Second, the reliance on retrospective data introduces potential biases, as improvements in diagnostic methods over time may reflect increased case detection rather than a true rise in incidence. Third, the COVID-19 pandemic significantly disrupted healthcare systems in Central Asia, delaying the diagnosis and treatment of chronic diseases like CE (34, 35). The reallocation of medical and veterinary resources during the pandemic, coupled with interruptions in routine public health campaigns, likely exacerbated gaps in disease prevention and control efforts, particularly in rural areas with higher zoonotic transmission risks. Additionally, socioeconomic disruptions may have intensified reliance on informal livestock practices, further complicating CE control (13, 33, 36). While broader factors such as climate change and traditional agricultural practices may influence CE epidemiology, these aspects were beyond the scope of our analysis. Future research could explore these factors to provide a more comprehensive understanding of CE transmission dynamics and refine public health strategies.

These findings emphasize the need for tailored public health interventions in Central Asia, particularly improving healthcare access in rural areas, strengthening disease surveillance systems, and implementing educational campaigns targeting high-risk groups, including males, children, and younger cohorts. Additionally, addressing gender-specific occupational risks and promoting safe livestock practices are crucial for reducing exposure. Sustained investment in regional collaboration and public health infrastructure is essential for mitigating the CE burden effectively and ensuring the long-term success of these interventions.

## 5 Conclusion

This study provides a comprehensive analysis of cystic echinococcosis (CE) trends in Central Asia over the past 30 years using the latest GBD 2021 data. The findings indicate that, despite some improvements, CE remains a significant public health challenge in Central Asia, with men experiencing significantly higher incidence rates than women. These results underscore the need for gender-specific prevention strategies and highlight substantial disparities among Central Asian countries in disease burden and management effectiveness. Public health policies should be tailored to consider gender, age, and national specifics to enhance their impact. Continued monitoring of CE trends and the development of targeted prevention strategies within diverse socioeconomic contexts are essential to reduce the disease burden in this high-risk region.

## Data Availability

The datasets presented in this study can be found in online repositories. The names of the repository/repositories and accession number(s) can be found at: http://ghdx.healthdata.org/gbd-results-tool.
