# Supplementary material for: Trends in incidence, mortality, and DALYs of cystic echinococcosis in Central Asia from 1992 to 2021: an age-period-cohort analysis
Source: Front Public Health. 2025 Jan 23;12:1504481. doi: 10.3389/fpubh.2024.1504481 (PMC11826808; doi:10.3389/fpubh.2024.1504481)
Supplement: Supplementary file 1 [file Data_Sheet_1.docx]

Supplementary Material

# Supplementary Data

Supplementary Material should be uploaded separately on submission. Please include any supplementary data, figures and or tables.

Supplementary material is not typeset so please ensure that all information is clearly presented, the appropriate caption is included in the file and not in the manuscript, and that the style conforms to the rest of the article.

# Supplementary Figures and Tables

For more information on Supplementary Material and for details on the different file types accepted, please see [here](https://www.frontiersin.org/guidelines/author-guidelines#supplementary-material).

## Supplementary Figures


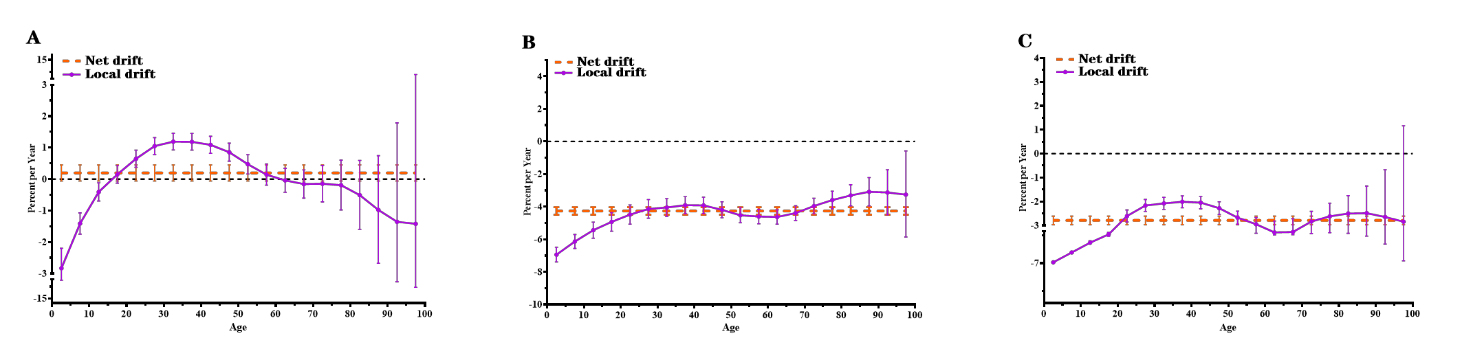


**Figure S1.** Local and net drift values for incidence, mortality, and DALY rates of CE in global, 1992–2021. **(A)** Incidence rate; **(B)** Mortality rate; **(C)** DALY rate.

Note: Net drift (dotted line) represents the overall annual percentage change across all age groups during the study period. Local drift (continuous line) represents the annual percentage change specific to each age group. A trend is considered statistically significant if its 95% confidence interval (CI) does not include 0.

Abbreviations: DALY, disability-adjusted life year; CE, cystic echinococcosis.


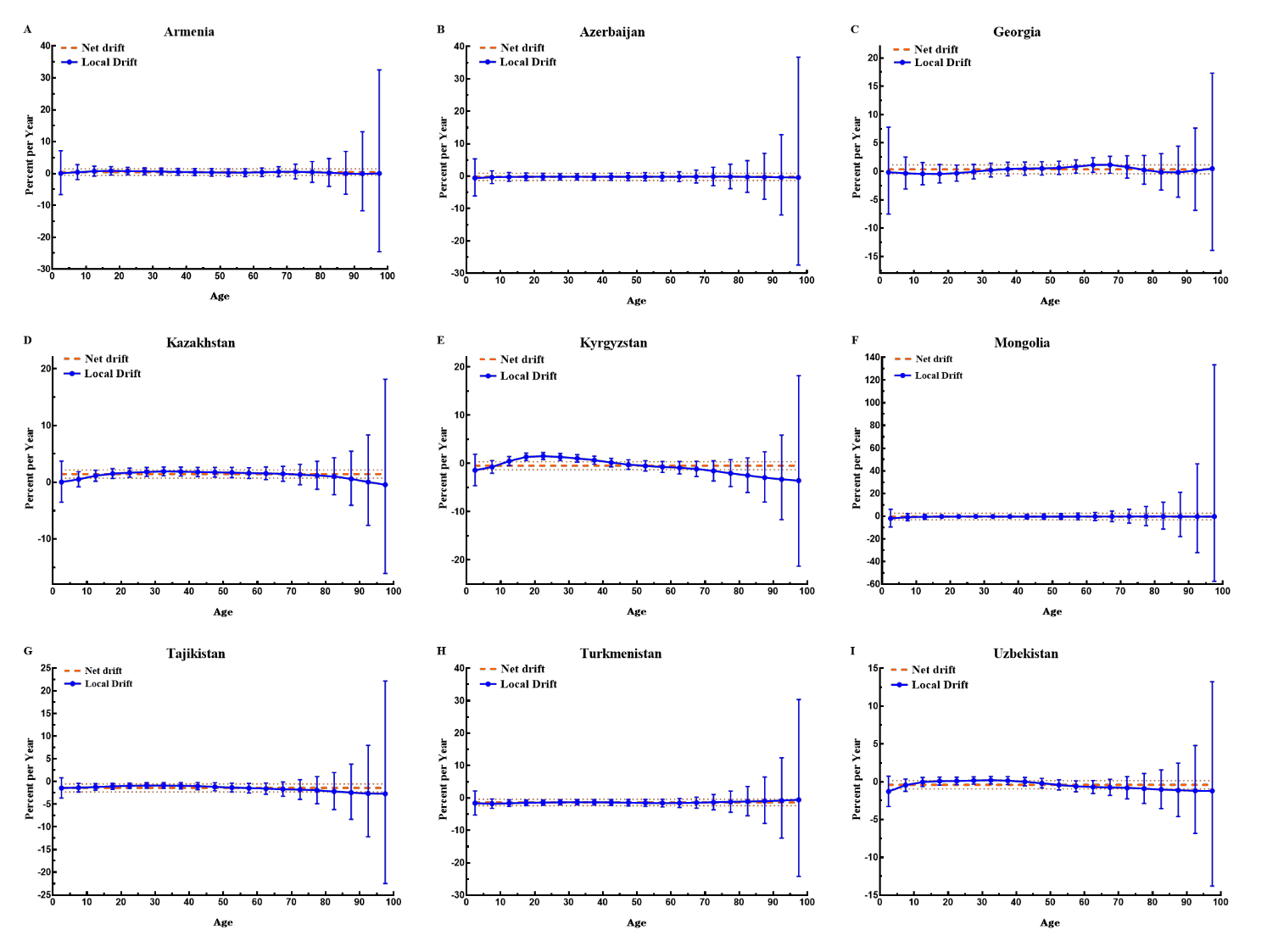


**Figure S2.** Local and net drift values for incidence of CE in nine Central Asian countries, 1992–2021. **(A)** Armenia; **(B)** Azerbaijan; **(C)** Georgia; **(D)** Kazakhstan; **(E)** Kyrgyzstan; **(F)** Monglia; **(G)** Tajikistan; **(H)** Turkmenistan; **(I)** Uzbekistan.

Note: Net drift (dotted line) represents the overall annual percentage change across all age groups during the study period. Local drift (continuous line) represents the annual percentage change specific to each age group. A trend is considered statistically significant if its 95% confidence interval (CI) does not include 0.

Abbreviations: CE, cystic echinococcosis.


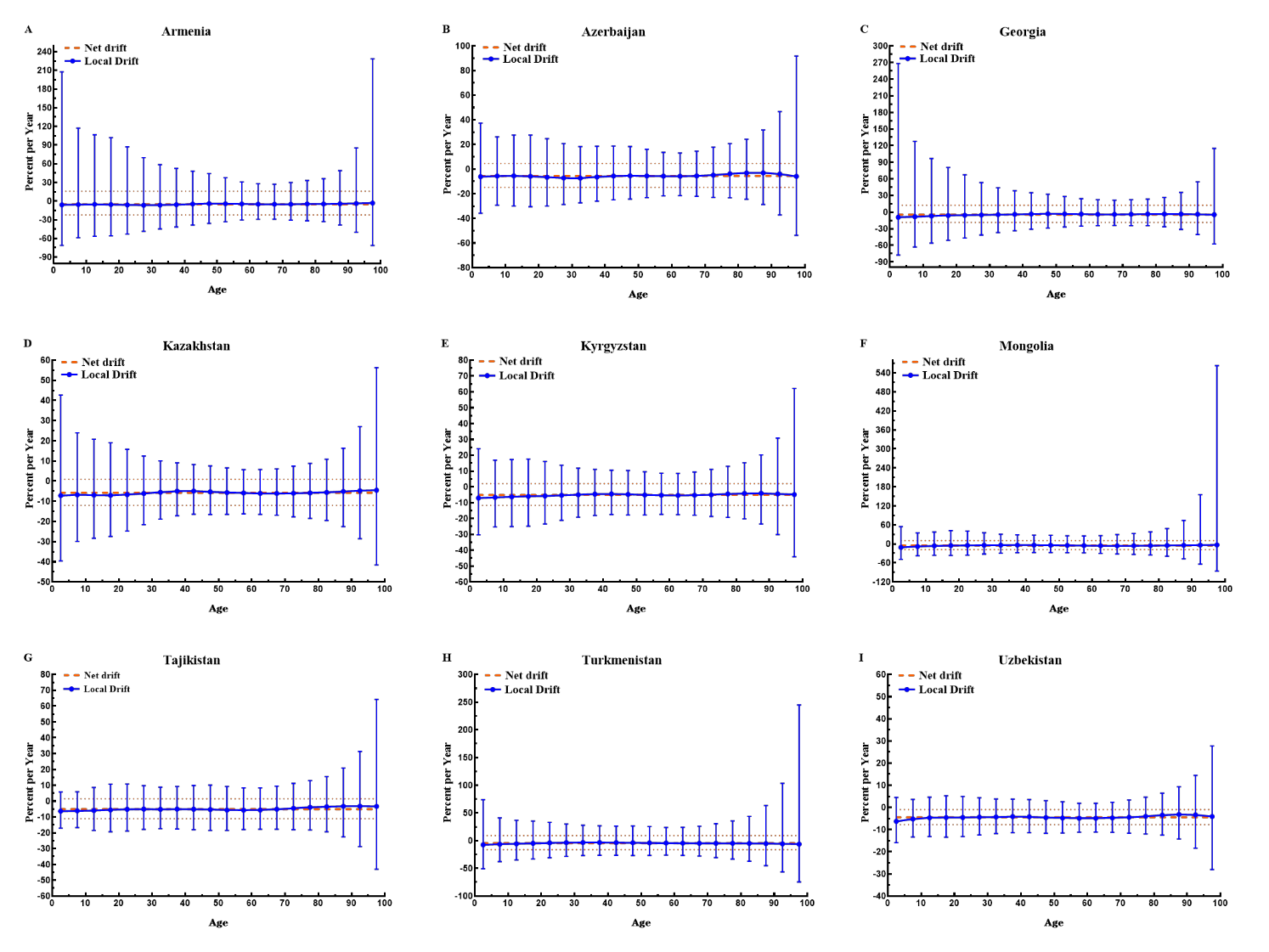


**Figure S3.** Local and net drift values for mortality of CE in nine Central Asian countries, 1992–2021. **(A)** Armenia; **(B)** Azerbaijan; **(C)** Georgia; **(D)** Kazakhstan; **(E)** Kyrgyzstan; **(F)** Monglia; **(G)** Tajikistan; **(H)** Turkmenistan; **(I)** Uzbekistan.

Note: Net drift (dotted line) represents the overall annual percentage change across all age groups during the study period. Local drift (continuous line) represents the annual percentage change specific to each age group. A trend is considered statistically significant if its 95% confidence interval (CI) does not include 0.

Abbreviations: CE, cystic echinococcosis.


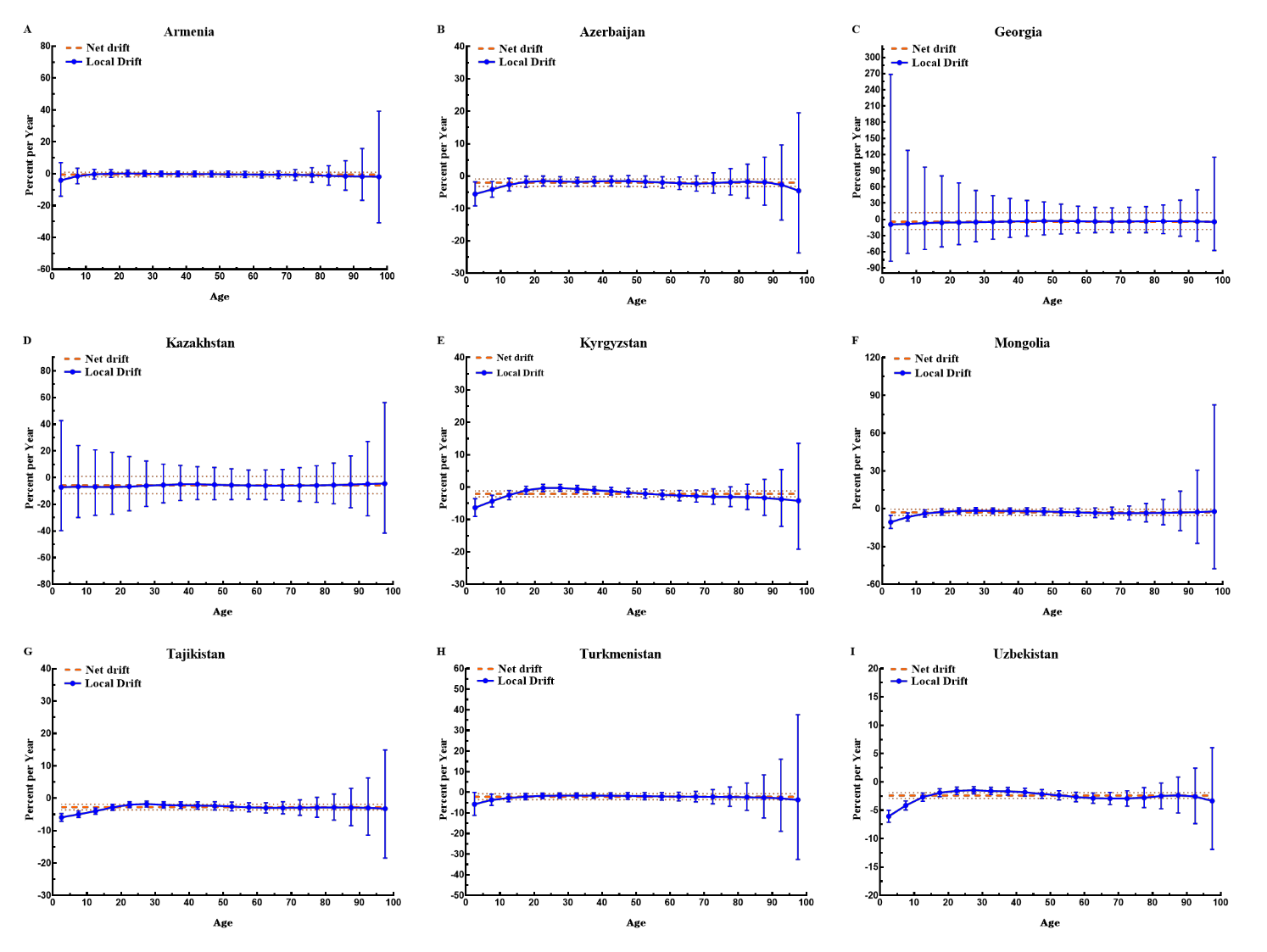


**Figure S4.** Local and net drift values for DALY rates of CE in nine Central Asian countries, 1992–2021. **(A)** Armenia; **(B)** Azerbaijan; **(C)** Georgia; **(D)** Kazakhstan; **(E)** Kyrgyzstan; **(F)** Monglia; **(G)** Tajikistan; **(H)** Turkmenistan; **(I)** Uzbekistan.

Note: Net drift (dotted line) represents the overall annual percentage change across all age groups during the study period. Local drift (continuous line) represents the annual percentage change specific to each age group. A trend is considered statistically significant if its 95% confidence interval (CI) does not include 0.

Abbreviations: DALY, disability-adjusted life year; CE, cystic echinococcosis.


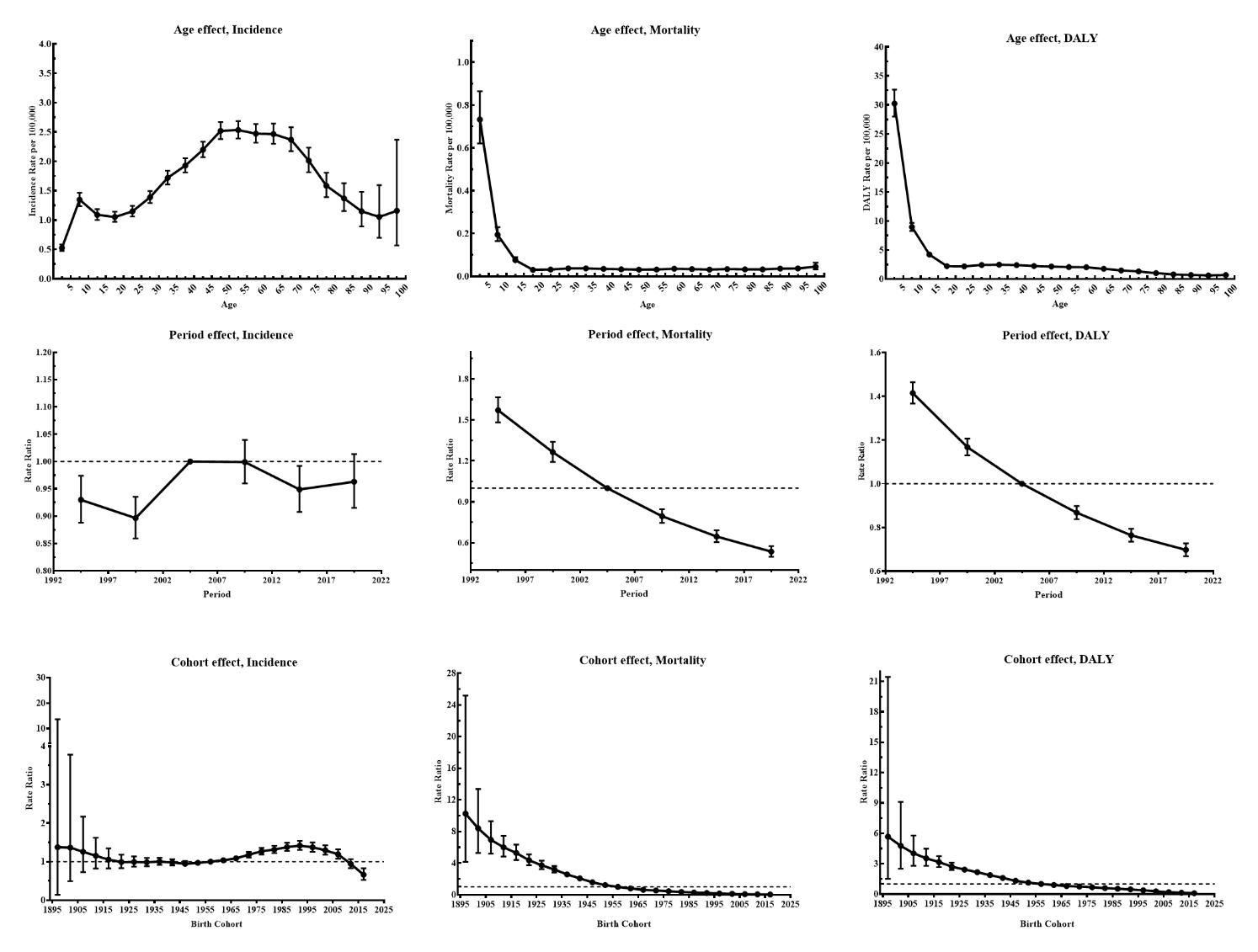


**Figure S5.** Parameter estimates of age, period, and cohort effects on incidence, mortality, and DALY rates of CE in global, 1992–2021. **(A)** Longitudinal age curves of CE incidence with corresponding 95% CI; **(B)** Longitudinal age curves of CE mortality with corresponding 95% CI; **(C)** Longitudinal age curves of CE DALY with corresponding 95% CI; **(D)** RR of each period compared with the reference (2002–2006), adjusted for age and nonlinear cohort effects, for incidence, with corresponding 95% CI; **(E)** RR of each period compared with the reference (2002–2006), adjusted for age and nonlinear cohort effects, for mortality, with corresponding 95% CI; **(F) )** RR of each period compared with the reference (2002–2006), adjusted for age and nonlinear cohort effects, for DALY, with corresponding 95% CI; **(G)** RR of each cohort compared with the reference (cohort 1955–1959), adjusted for age and nonlinear period effects, for incidence, with corresponding 95% CI; **(H)** RR of each cohort compared with the reference (cohort 1955–1959), adjusted for age and nonlinear period effects, for mortality, with corresponding 95% CI; **(I)** RR of each cohort compared with the reference (cohort 1955–1959), adjusted for age and nonlinear period effects, for DALY, with corresponding 95% CI;

Abbreviations: DALY, disability-adjusted life year; CE, cystic echinococcosis; CI, confidence interval; RR, relative risk.


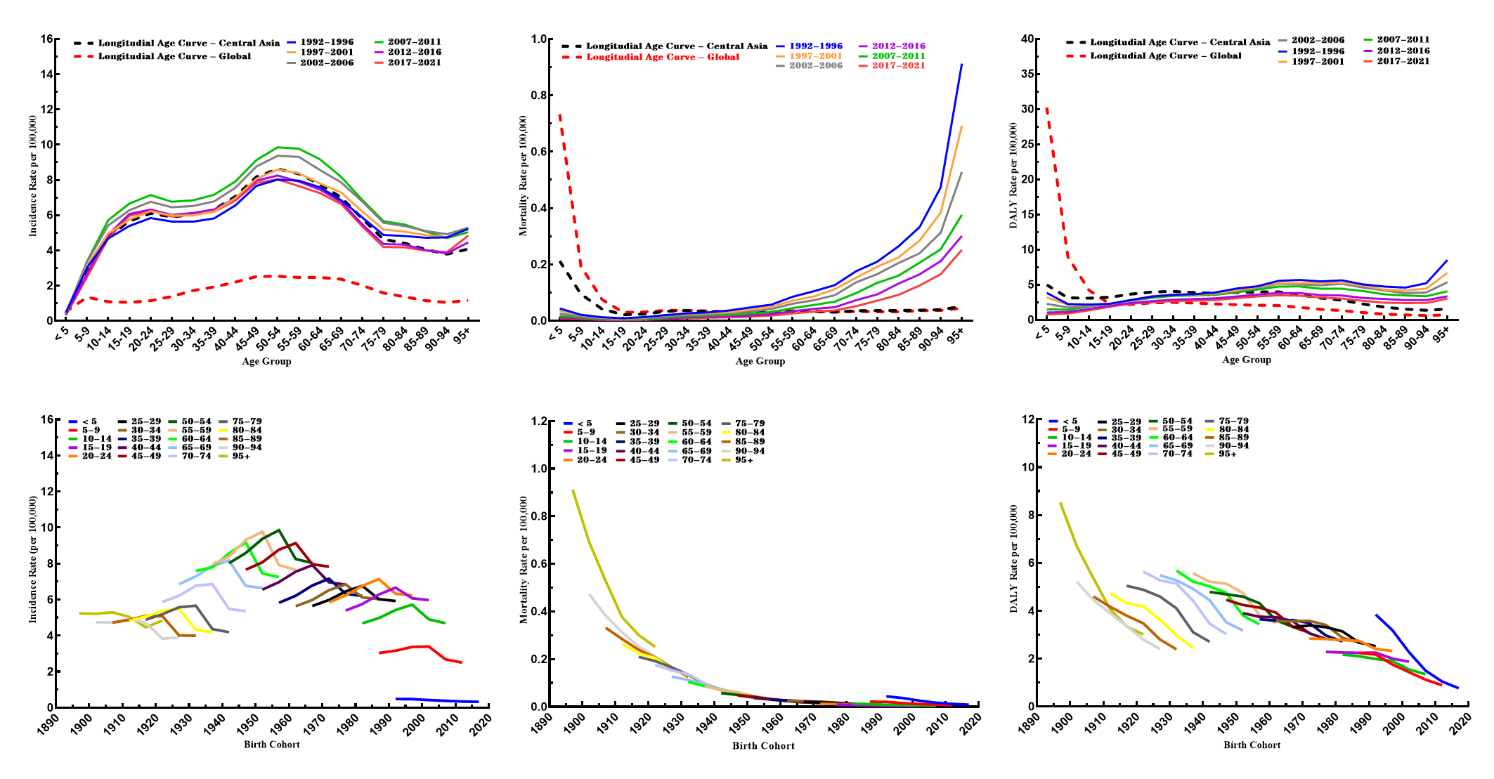
 **Figure S6.** Age-specific and cohort-specific incidence, mortality, and DALY rates of CE in Central Asia and globally, 1992–2021. **(A)** Age-specific incidence rates; **(B)** Age-specific mortality rates; **(C)** Age-specific DALY rates; **(D)** Cohort-specific incidence rates; **(E)** Cohort-specific mortality rates; **(F)** Cohort-specific DALY rates;

Note: A through C, Survey years were arranged into consecutive 5-year periods from 1992 to 1996 (median, 1994), 1997 to 2001 (median, 1999), 2002 to 2006 (median, 2004), 2007 to 2011 (median, 2009), 2012 to 2016 (median, 2014), and 2017 to 2021 (median, 2019). Longitudinal age curves were estimated by age-period-cohort model and indicated the expected age-specific rate of CE incidence, mortality, and DALY rate. D through F, The CE incidence, mortality, and DALY rate data were arranged into 26 consecutive birth cohorts, including those born from 1897 to 1901 (median, 1899) to 2017 to 2021 (median, 2019), and successive 5-year age intervals from 0 to 4 years (median, 2 years) to 95 to 99(median, 97 years) years of age.

Abbreviations: CE, cystic echinococcosis; DALY, disability-adjusted life year.

**
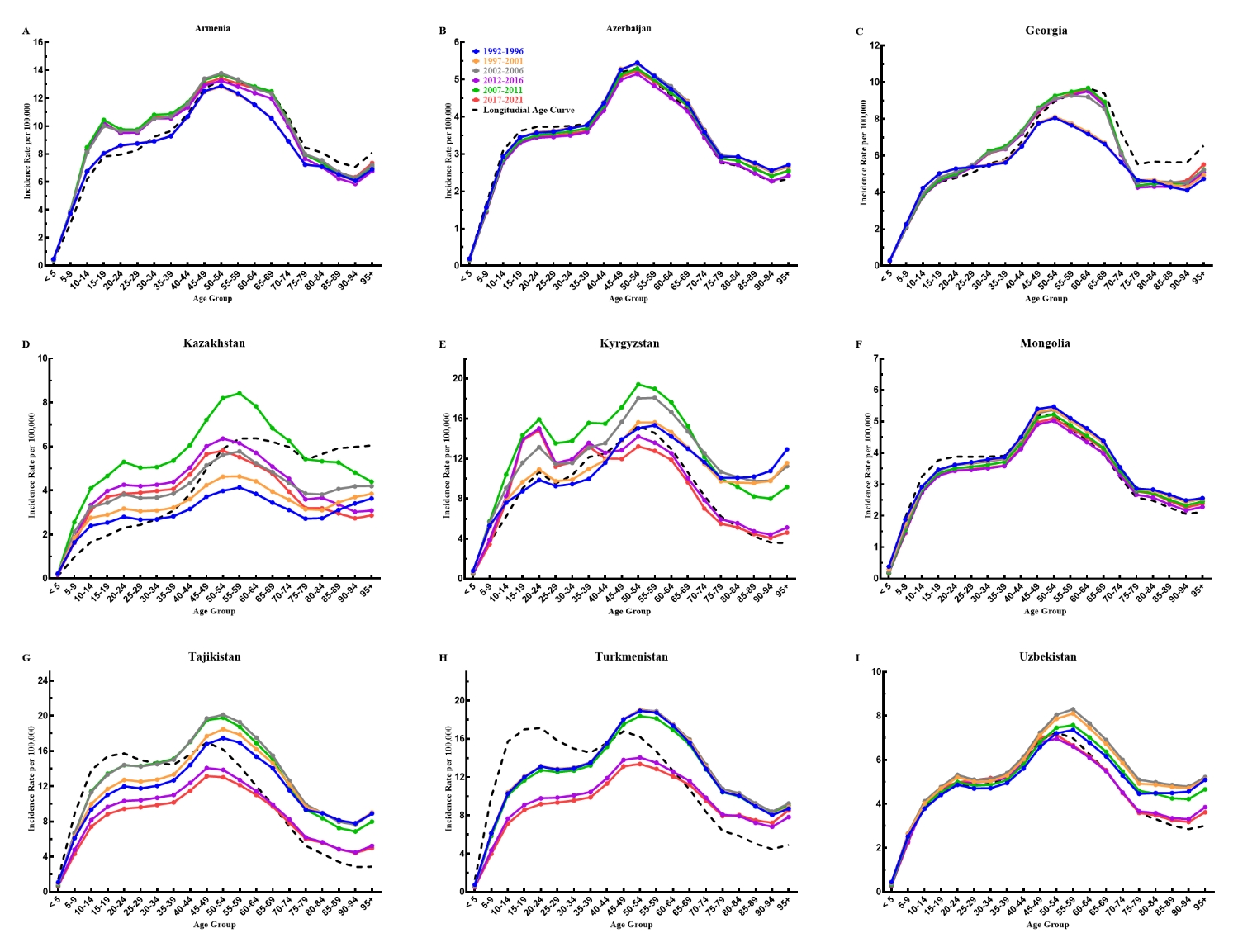
**

**Figure S7.** Age-specific incidence rates of CE in nine Central Asian countries from 1992 to 2021. **(A)** Armenia**; (B)** Azerbaijan**; (C)** Georgia; **(D)** Kazakhstan; **(E)** Kyrgyzstan; **(F)** Mongolia; **(G)** Tajikistan; **(H)** Turkmenistan; **(I)** Uzbekistan.

Abbreviations: CE, cystic echinococcosis; DALY, disability-adjusted life year.


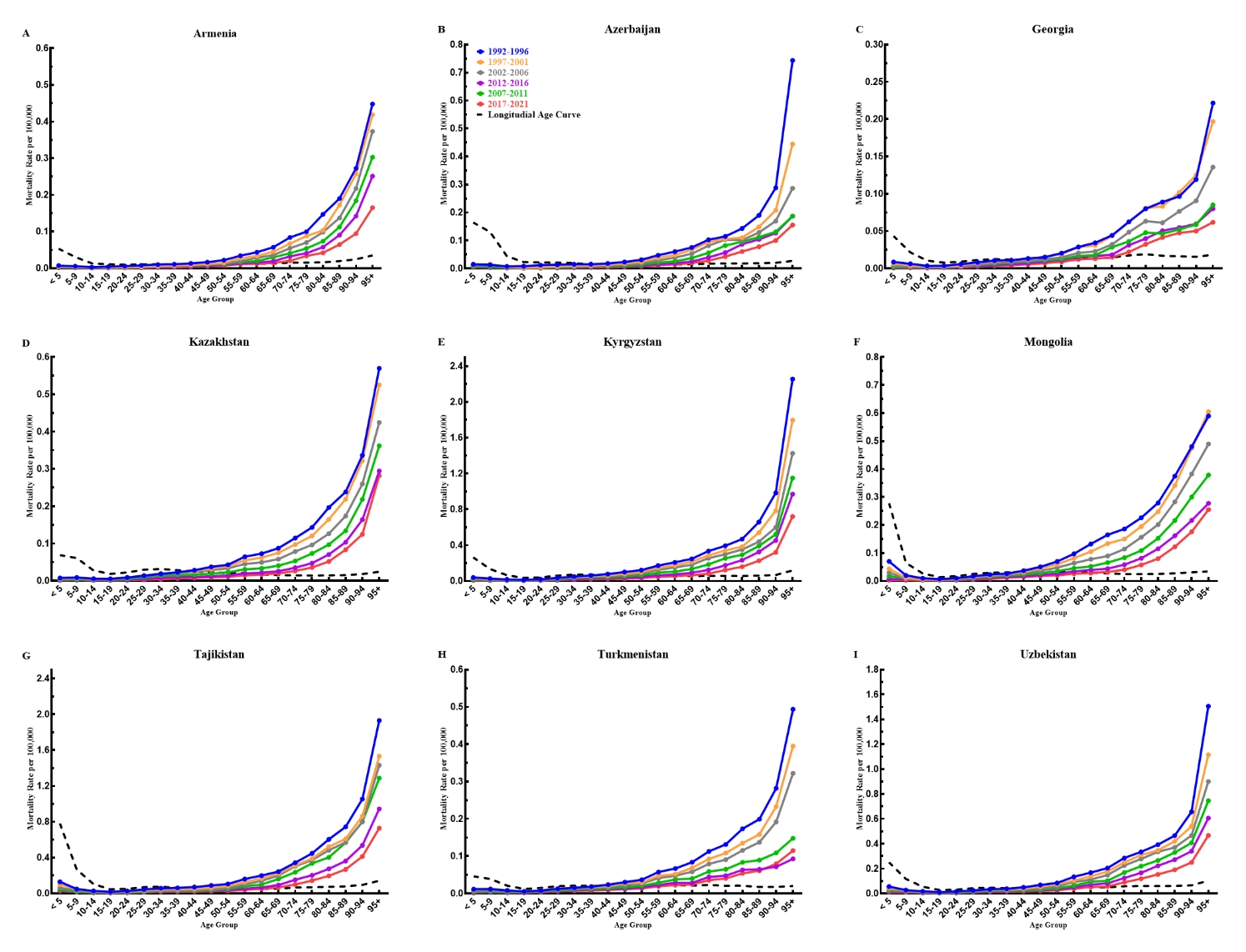


**Figure S8.** Age-specific mortality rates of CE in nine Central Asian countries from 1992 to 2021. **(A)** Armenia; **(B)** Azerbaijan; **(C)** Georgia; **(D)** Kazakhstan; **(E)** Kyrgyzstan; **(F)** Mongolia; **(G)** Tajikistan; **(H)** Turkmenistan; **(I)** Uzbekistan.

Abbreviations: CE, cystic echinococcosis; DALY, disability-adjusted life year.


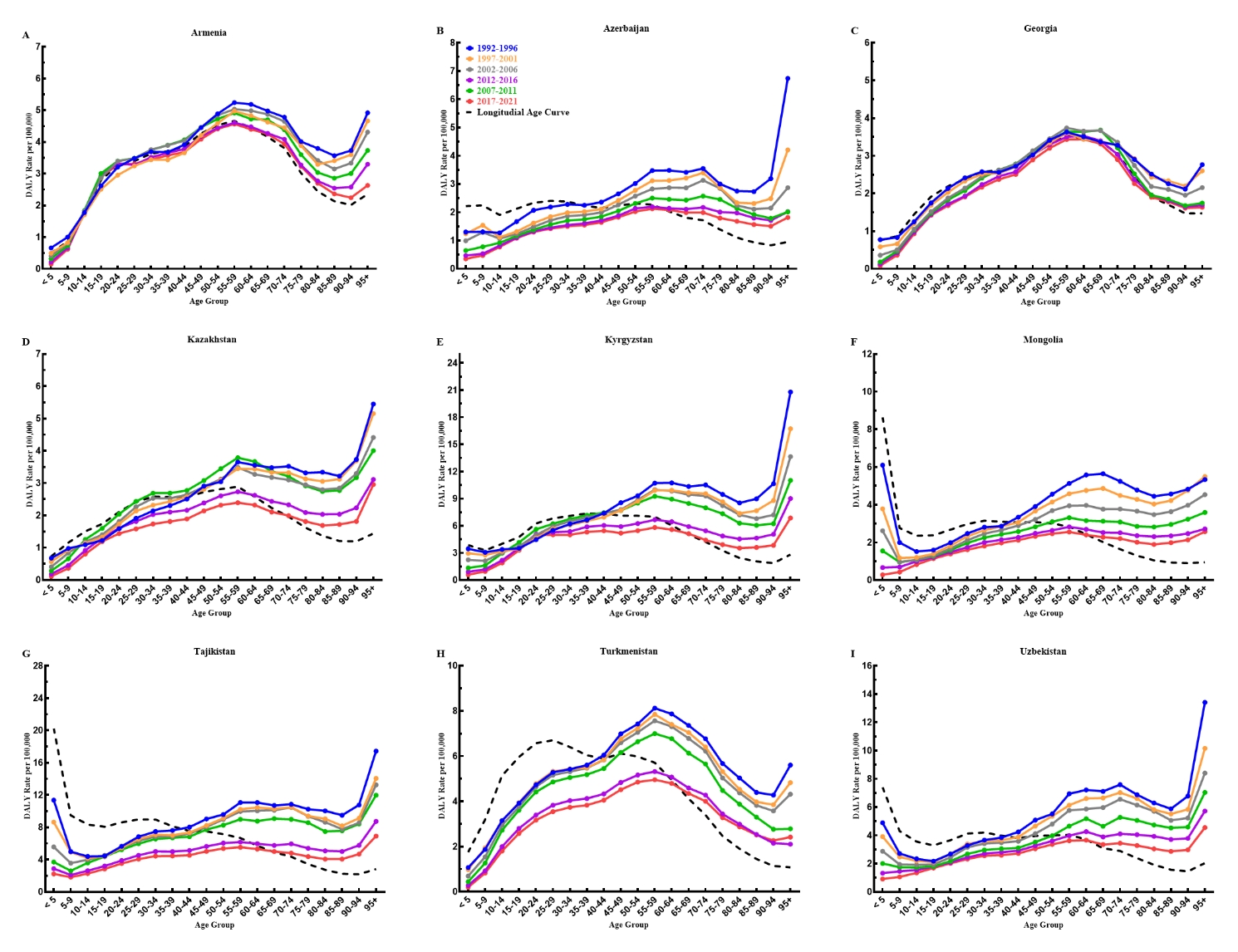


**Figure S9.** Age-specific DALY rates of CE in nine Central Asian countries from 1992 to 2021. **(A)** Armenia; **(B)** Azerbaijan; **(C)** Georgia; **(D)** Kazakhstan; **(E)** Kyrgyzstan; **(F)** Mongolia; **(G)** Tajikistan; **(H)** Turkmenistan; **(I)** Uzbekistan.

Abbreviations: CE, cystic echinococcosis; DALY, disability-adjusted life year.


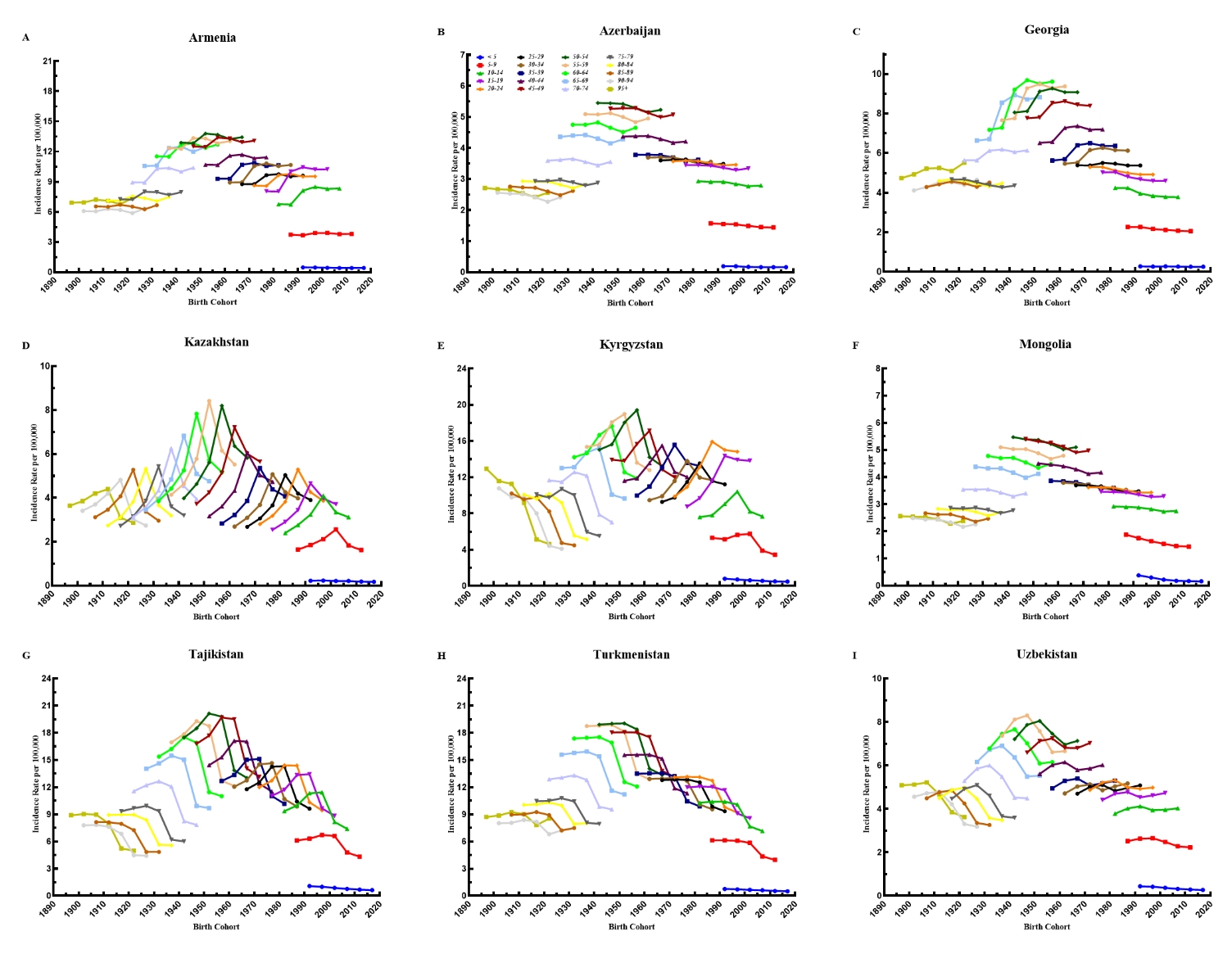


**Figure S11.** Cohort-specific incidence rates of CE in nine Central Asian countries from 1992 to 2021. **(A)** Armenia; **(B)** Azerbaijan; **(C)** Georgia; **(D)** Kazakhstan; **(E)** Kyrgyzstan; **(F)** Mongolia; **(G)** Tajikistan; **(H)** Turkmenistan; **(I)** Uzbekistan.

Abbreviations: CE, cystic echinococcosis; ASIR, age-standardized incidence rate; ASMR, age-standardized mortality rate; DALY, disability-adjusted life year; ASDR, age-standardized DALY rate.


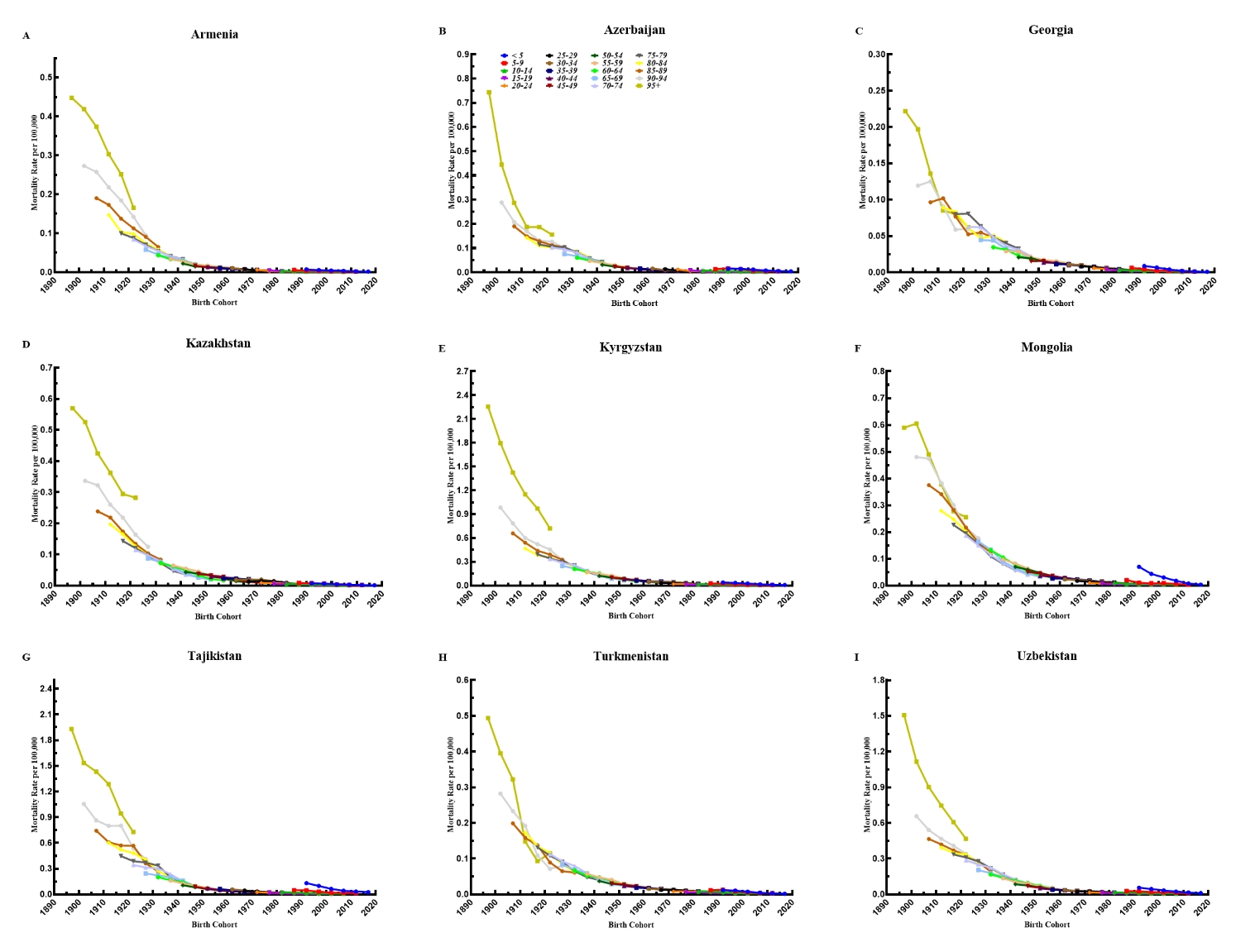


**Figure S12.** Cohort-specific mortality rates of CE in nine Central Asian countries from 1992 to 2021. **(A)** Armenia; **(B)** Azerbaijan; **(C)** Georgia; **(D)** Kazakhstan; **(E)** Kyrgyzstan; **(F)** Mongolia; **(G)** Tajikistan; **(H)** Turkmenistan; **(I)** Uzbekistan.

Abbreviations: CE, cystic echinococcosis.


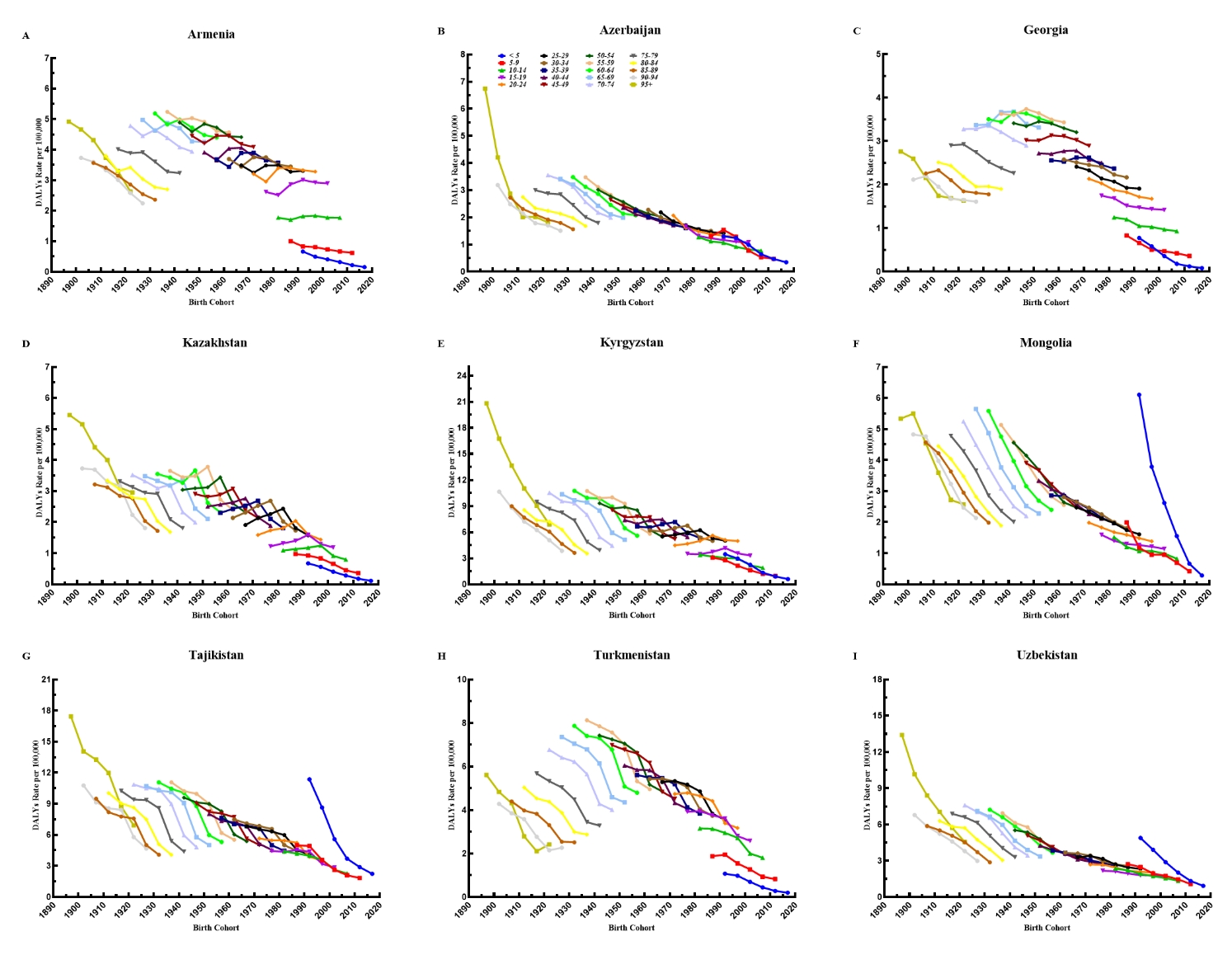


**Figure S13.** Cohort-specific DALY rates of CE in nine Central Asian countries from 1992 to 2021. **(A)** Armenia; **(B)** Azerbaijan; **(C)** Georgia; **(D)** Kazakhstan; **(E)** Kyrgyzstan; **(F)** Mongolia; **(G)** Tajikistan; **(H)** Turkmenistan; **(I)** Uzbekistan.

Abbreviations: CE, cystic echinococcosis; DALY, disability-adjusted life year.

## Supplementary Tables

**Table S1.** Incidence, mortality, and DALY number for CE by gender, globally and in Central Asian countries, for 1992 and 2021, with trends over 1992–2021.

| Item | Incidence number (95% UI) | | | Mortality number (95% UI) | | | DALYs (95% UI) | | |
| --- | --- | --- | --- | --- | --- | --- | --- | --- | --- |
|  | 1992 | 2021 | Percent Change (% [95% UI]) | 1992 | 2021 | Percent Change (% [95% UI]) | 1992 | 2021 | Percent Change (% [95% UI]) |
| Male | | | | | | | | | |
| Global | 41795 (34488, 51753) | 64830 (52416, 80760) | 55.11 (51.98, 56.05) | 1879 (1420, 2428) | 727 (445, 1022) | -61.31 (-68.68, -57.89) | 126310 (97069, 161218) | 53027 (37127, 70035) | -58.02 (-61.75, -56.56) |
| Armenia | 124 (99, 154) | 126 (102, 155) | 2.07 (3.1, 0.68) | 0 (0, 1) | 0 (0, 0) | -67.81 (-71.03, -67.14) | 53 (34, 74) | 42 (26, 62) | -21.17 (-25.17, -15.69) |
| Azerbaijan | 119 (94, 149) | 186 (147, 227) | 55.33 (55.88, 52.21) | 1 (0, 2) | 0 (0, 1) | -64.12 (-66.4, -64.26) | 91 (51, 131) | 76 (48, 110) | -16 (-5.07, -15.98) |
| Georgia | 121 (95, 146) | 85 (71, 100) | -29.53 (-25.35, -31.53) | 1 (0, 1) | 0 (0, 0) | -71.1 (-74.61, -70.49) | 61 (38, 87) | 32 (20, 47) | -47.55 (-46.15, -46.45) |
| Kazakhstan | 203 (185, 221) | 354 (279, 436) | 74.26 (50.69, 97.41) | 2 (1, 4) | 1 (0, 1) | -66.91 (-69.94, -64.27) | 169 (101, 240) | 142 (88, 209) | -16.28 (-12.02, -12.93) |
| Kyrgyzstan | 203 (187, 219) | 319 (250, 410) | 56.96 (34.09, 87.22) | 2 (1, 3) | 1 (0, 1) | -56.37 (-62.72, -55.27) | 142 (87, 202) | 131 (83, 181) | -7.99 (-3.58, -10.05) |
| Mongolia | 35 (27, 45) | 52 (41, 65) | 47.96 (51.13, 45.9) | 0 (0, 1) | 0 (0, 0) | -55.36 (-57, -59.49) | 38 (16, 72) | 26 (16, 37) | -31.13 (0.61, -48.66) |
| Tajikistan | 258 (222, 304) | 441 (350, 548) | 70.7 (57.57, 79.82) | 2 (1, 4) | 1 (0, 2) | -53.97 (-62.07, -52.47) | 222 (118, 332) | 194 (120, 287) | -12.51 (2.3, -13.55) |
| Turkmenistan | 216 (169, 268) | 238 (189, 297) | 10.22 (11.74, 10.82) | 0 (0, 1) | 0 (0, 0) | -34.54 (-43.56, -35.4) | 84 (52, 123) | 84 (52, 125) | -0.11 (1.44, 2.11) |
| Uzbekistan | 481 (420, 547) | 822 (657, 1035) | 70.99 (56.41, 89.14) | 6 (3, 9) | 3 (1, 5) | -46.91 (-56.09, -43.62) | 470 (295, 645) | 406 (252, 563) | -13.69 (-14.51, -12.73) |
| Female | | | | | | | | | |
| Global | 50541 (41933, 62225) | 83691 (67299, 103238) | 65.59 (60.49, 65.91) | 1698 (1256, 2245) | 637 (369, 874) | -62.5 (-70.65, -61.08) | 118603 (92346, 154421) | 52045 (37561, 69506) | -56.12 (-59.33, -54.99) |
| Armenia | 149 (120, 184) | 166 (132, 202) | 11.14 (9.82, 9.66) | 0 (0, 0) | 0 (0, 0) | -67.28 (-73.87, -65.19) | 54 (34, 78) | 52 (31, 76) | -3.39 (-8.31, -2.1) |
| Azerbaijan | 120 (94, 149) | 178 (139, 218) | 49.07 (47.49, 46.39) | 1 (0, 1) | 0 (0, 0) | -63.94 (-70.74, -61.96) | 66 (44, 91) | 68 (40, 99) | 2.48 (-8.13, 8.96) |
| Georgia | 164 (130, 199) | 131 (111, 157) | -19.85 (-14.8, -21.3) | 0 (0, 1) | 0 (0, 0) | -74.65 (-77.65, -73.28) | 66 (44, 96) | 44 (28, 65) | -33.29 (-36.82, -32.11) |
| Kazakhstan | 178 (162, 193) | 337 (264, 412) | 89.6 (62.7, 113.24) | 2 (1, 3) | 0 (0, 1) | -72.31 (-76.06, -71.35) | 118 (83, 164) | 124 (75, 178) | 5.31 (-10.21, 9.04) |
| Kyrgyzstan | 166 (152, 178) | 332 (263, 414) | 99.97 (73.31, 132.36) | 1 (1, 2) | 0 (0, 1) | -68.15 (-70.25, -68.05) | 105 (68, 149) | 119 (77, 179) | 13.1 (13.67, 19.56) |
| Mongolia | 33 (26, 42) | 51 (40, 63) | 56.25 (56.74, 50.23) | 0 (0, 1) | 0 (0, 0) | -76.73 (-73.19, -79.47) | 34 (16, 63) | 21 (13, 30) | -39.2 (-14.94, -53.12) |
| Tajikistan | 210 (180, 249) | 375 (298, 465) | 78.35 (65.21, 86.76) | 2 (1, 3) | 1 (0, 1) | -59.04 (-60.03, -58.84) | 157 (90, 245) | 152 (95, 222) | -3.15 (5.2, -9.68) |
| Turkmenistan | 189 (153, 224) | 200 (159, 244) | 5.76 (3.87, 8.76) | 0 (0, 1) | 0 (0, 0) | -53.54 (-62.86, -52.52) | 73 (48, 104) | 67 (39, 100) | -7.51 (-17.94, -4.15) |
| Uzbekistan | 397 (351, 449) | 727 (582, 902) | 83.26 (65.63, 100.81) | 5 (3, 8) | 2 (1, 4) | -53.1 (-60.42, -49.99) | 357 (234, 506) | 327 (208, 469) | -8.43 (-11.43, -7.35) |

Abbreviation:UI, uncertainty interval;

**Table S2.** Age-standardized incidence, mortality, and DALY rates for CE by gender, globally and in Central Asian countries for 1992 and 2021, with trends over 1992–2021.

| Item | ASIR, per 100,000 (95% UI) | | | ASMR, per 100,000 (95% UI) | | | ASDR, per 100,000 (95% UI) | | |
| --- | --- | --- | --- | --- | --- | --- | --- | --- | --- |
|  | 1992 | 2021 | AAPC (%, 95% CI) | 1992 | 2021 | AAPC (%, 95% CI) | 1992 | 2021 | AAPC (%, 95% CI) |
| Male | | | | | | | | | |
| Global | 1.55 (1.3 , 1.89) | 1.59 (1.3 , 1.99) | 0.08 (0.03 , 0.13) | 0.07 (0.06 , 0.09) | 0.02 (0.01 , 0.03) | -4.68 (-4.74 , -4.62) | 4.43 (3.42 , 5.54) | 1.34 (0.93 , 1.79) | -4.05 (-3.97 , -4.13) |
| Armenia | 7.6 (6.15 , 9.32) | 8.21 (6.64 , 10.12) | 0.27 (0.16 , 0.39) | 0.03 (0.01 , 0.04) | 0.01 (0 , 0.01) | -4.71 (-5.2 , -4.23) | 3.28 (2.17 , 4.62) | 2.66 (1.6 , 3.97) | -0.7 (-1.01 , -0.38) |
| Azerbaijan | 3.48 (2.79 , 4.2) | 3.33 (2.66 , 4.05) | -0.16 (-0.18 , -0.14) | 0.03 (0.02 , 0.06) | 0.01 (0 , 0.01) | -5.11 (-5.56 , -4.66) | 2.61 (1.53 , 3.66) | 1.38 (0.88 , 1.97) | -2.19 (-2.51 , -1.87) |
| Georgia | 4.55 (3.64 , 5.51) | 4.58 (3.82 , 5.35) | 0.02 (0 , 0.05) | 0.02 (0.01 , 0.04) | 0.01 (0 , 0.01) | -3.48 (-4.29 , -2.66) | 2.31 (1.44 , 3.23) | 1.68 (1.08 , 2.46) | -1.05 (-1.27 , -0.83) |
| Kazakhstan | 2.72 (2.49 , 2.93) | 3.86 (3.07 , 4.68) | 1.19 (0.86 , 1.52) | 0.04 (0.02 , 0.06) | 0.01 (0 , 0.02) | -4.63 (-5.12 , -4.14) | 2.32 (1.44 , 3.18) | 1.55 (0.98 , 2.25) | -1.37 (-1.77 , -0.96) |
| Kyrgyzstan | 10.22 (9.42 , 10.95) | 9.56 (7.64 , 11.99) | -0.19 (-0.35 , -0.04) | 0.11 (0.05 , 0.18) | 0.03 (0.01 , 0.05) | -4.47 (-4.88 , -4.07) | 7.21 (4.61 , 9.95) | 4.03 (2.62 , 5.65) | -1.99 (-2.18 , -1.8) |
| Mongolia | 3.56 (2.85 , 4.34) | 3.26 (2.61 , 4.01) | -0.3 (-0.32 , -0.27) | 0.06 (0.02 , 0.11) | 0.02 (0.01 , 0.03) | -4.62 (-5.61 , -3.62) | 3.67 (1.83 , 6.06) | 1.66 (1.06 , 2.32) | -2.71 (-3.24 , -2.18) |
| Tajikistan | 10.94 (9.42 , 12.7) | 9.05 (7.32 , 11.02) | -0.67 (-0.88 , -0.46) | 0.11 (0.06 , 0.18) | 0.03 (0.01 , 0.05) | -4.64 (-4.92 , -4.37) | 8.28 (5.11 , 11.38) | 4 (2.59 , 5.8) | -2.47 (-2.69 , -2.26) |
| Turkmenistan | 13.06 (10.37 , 15.86) | 9.14 (7.31 , 11.26) | -1.2 (-1.39 , -1.01) | 0.03 (0.01 , 0.05) | 0.01 (0 , 0.02) | -3.55 (-4.3 , -2.79) | 5.19 (3.33 , 7.27) | 3.22 (2.02 , 4.84) | -1.65 (-1.89 , -1.4) |
| Uzbekistan | 5.11 (4.54 , 5.69) | 4.86 (3.89 , 5.97) | -0.16 (-0.24 , -0.09) | 0.08 (0.04 , 0.12) | 0.02 (0.01 , 0.04) | -4.26 (-4.62 , -3.89) | 4.81 (3.16 , 6.52) | 2.43 (1.5 , 3.35) | -2.38 (-2.54 , -2.22) |
| Female | | | | | | | | | |
| Global | 1.92 (1.6 , 2.33) | 2.05 (1.66 , 2.55) | 0.23 (0.19 , 0.27) | 0.06 (0.05 , 0.08) | 0.02 (0.01 , 0.02) | -4.88 (-4.96 , -4.8) | 4.25 (3.37 , 5.48) | 1.3 (0.92 , 1.77) | -4 (-3.93 , -4.07) |
| Armenia | 8.57 (6.96 , 10.49) | 9.58 (7.67 , 11.85) | 0.42 (0.33 , 0.51) | 0.02 (0.01 , 0.03) | 0 (0 , 0.01) | -5.25 (-5.47 , -5.03) | 3.08 (1.97 , 4.48) | 2.9 (1.75 , 4.33) | -0.17 (-0.32 , -0.03) |
| Azerbaijan | 3.25 (2.59 , 3.94) | 3.1 (2.45 , 3.76) | -0.15 (-0.2 , -0.09) | 0.02 (0.01 , 0.04) | 0 (0 , 0.01) | -4.99 (-5.27 , -4.71) | 1.8 (1.19 , 2.49) | 1.16 (0.71 , 1.71) | -1.51 (-1.65 , -1.38) |
| Georgia | 5.4 (4.26 , 6.55) | 5.93 (4.94 , 6.98) | 0.34 (0.27 , 0.4) | 0.01 (0 , 0.02) | 0 (0 , 0.01) | -4.46 (-5.29 , -3.62) | 2.12 (1.41 , 3.12) | 1.9 (1.19 , 2.83) | -0.41 (-0.64 , -0.17) |
| Kazakhstan | 2.16 (1.97 , 2.35) | 3.35 (2.63 , 4.09) | 1.51 (1.2 , 1.81) | 0.02 (0.01 , 0.04) | 0 (0 , 0.01) | -5.08 (-5.31 , -4.84) | 1.44 (1.01 , 2) | 1.22 (0.74 , 1.75) | -0.55 (-0.78 , -0.33) |
| Kyrgyzstan | 7.76 (7.13 , 8.29) | 9.77 (7.81 , 12.11) | 0.82 (0.69 , 0.95) | 0.07 (0.03 , 0.12) | 0.02 (0.01 , 0.03) | -5.04 (-5.34 , -4.74) | 4.85 (3.2 , 6.64) | 3.53 (2.29 , 5.26) | -1.09 (-1.25 , -0.93) |
| Mongolia | 3.35 (2.69 , 4.1) | 3.06 (2.41 , 3.74) | -0.31 (-0.34 , -0.28) | 0.05 (0.02 , 0.09) | 0.01 (0 , 0.01) | -6.39 (-6.79 , -5.98) | 3.23 (1.78 , 5.3) | 1.25 (0.82 , 1.76) | -3.23 (-3.43 , -3.04) |
| Tajikistan | 8.81 (7.59 , 10.39) | 7.86 (6.3 , 9.61) | -0.37 (-0.54 , -0.2) | 0.09 (0.04 , 0.14) | 0.02 (0.01 , 0.04) | -4.64 (-4.88 , -4.4) | 6.09 (4.08 , 9.04) | 3.25 (2.04 , 4.61) | -2.12 (-2.28 , -1.96) |
| Turkmenistan | 11.08 (8.96 , 12.91) | 7.92 (6.33 , 9.66) | -1.12 (-1.32 , -0.92) | 0.02 (0.01 , 0.04) | 0.01 (0 , 0.01) | -4.31 (-4.88 , -3.74) | 4.34 (2.83 , 6.15) | 2.68 (1.57 , 3.97) | -1.66 (-1.92 , -1.39) |
| Uzbekistan | 4.08 (3.65 , 4.53) | 4.21 (3.43 , 5.17) | 0.12 (0.07 , 0.17) | 0.06 (0.03 , 0.09) | 0.02 (0.01 , 0.03) | -4.4 (-4.75 , -4.06) | 3.52 (2.4 , 4.72) | 1.91 (1.22 , 2.74) | -2.08 (-2.31 , -1.85) |

Abbreviation: ASIR, age-standardized incidence rate; ASMR, age-standardized mortality rate; ASDR, age-standardized DALY rate; CI, confidence interval; UI, uncertainty interval; AAPC, average annual percent change presented for full period.

**Table S3**. Trends in age-standardized incidence, mortality, and DALY rates of cystic echinococcosis in Central Asia and globally from 1992 to 2021, analyzed using joinpoint regression.

| **Factors** | **Trend 1** | | **Trend 2** | | **Trend 3** | | **Trend 4** | | **Trend 5** | | **Trend 6** | |
| --- | --- | --- | --- | --- | --- | --- | --- | --- | --- | --- | --- | --- |
|  | **Year** | **APC (95% CI)** | **Year** | **APC (95% CI)** | **Year** | **APC (95% CI)** | **Year** | **APC (95% CI)** | **Year** | **APC (95% CI)** | **Year** | **APC (95% CI)** |
| Age-standardized Incidence rate | | | | | | | | | | | | |
| Armenia | 1992-2000 | -0.21 (-0.34 , -0.07) | 2000-2005 | 3.45 (3.05 , 3.85) | 2005-2014 | -0.68 (-0.82 , -0.55) | 2014-2021 | 0.33 (0.16 , 0.49) |  |  |  |  |
| Azerbaijan | 1992-1994 | -0.51 (-0.81 , -0.21) | 1994-2005 | 0.06 (0.03 , 0.08) | 2005-2012 | -0.68 (-0.73 , -0.62) | 2012-2015 | -0.46 (-0.77 , -0.16) | 2015-2019 | 0.63 (0.48 , 0.79) | 2019-2021 | -0.19 (-0.51 , 0.12) |
| Georgia | 1992-1996 | -0.31 (-0.45 , -0.17) | 1996-2001 | 0.43 (0.29 , 0.58) | 2001-2004 | 2.62 (2.21 , 3.04) | 2004-2007 | -0.01 (-0.37 , 0.35) | 2007-2014 | -0.46 (-0.52 , -0.4) | 2014-2021 | 0.09 (0.05 , 0.14) |
| Kazakhstan | 1992-2004 | 2.91 (2.75 , 3.06) | 2004-2010 | 7.85 (7.26 , 8.45) | 2010-2015 | -8.37 (-9.47 , -7.25) | 2015-2021 | 0.48 (-0.64 , 1.6) |  |  |  |  |
| Kyrgyzstan | 1992-1995 | 1.95 (1.62 , 2.27) | 1995-2000 | 0.13 (-0.09 , 0.35) | 2000-2005 | 3.87 (3.61 , 4.14) | 2005-2010 | 2.38 (2.04 , 2.71) | 2010-2015 | -6.42 (-6.85 , -5.98) | 2015-2021 | 0.83 (0.52 , 1.14) |
| Mongolia | 1992-1994 | -0.52 (-0.7 , -0.34) | 1994-2006 | -0.23 (-0.24 , -0.22) | 2006-2012 | -0.84 (-0.88 , -0.8) | 2012-2015 | -0.66 (-0.84 , -0.48) | 2015-2019 | 0.62 (0.53 , 0.71) | 2019-2021 | -0.23 (-0.41 , -0.04) |
| Tajikistan | 1992-1994 | 7.43 (6.22 , 8.66) | 1994-2001 | 0.37 (0.17 , 0.56) | 2001-2005 | 3.4 (2.77 , 4.04) | 2005-2011 | -1.25 (-1.52 , -0.97) | 2011-2014 | -12.3 (-13.46 , -11.12) | 2014-2021 | 0.1 (-0.1 , 0.3) |
| Turkmenistan | 1992-2007 | 0.03 (-0.03 , 0.09) | 2007-2011 | -1.44 (-2.12 , -0.76) | 2011-2014 | -9.24 (-10.5 , -7.96) | 2014-2021 | 0.13 (-0.07 , 0.34) |  |  |  |  |
| Uzbekistan | 1992-1995 | -0.96 (-1.23 , -0.69) | 1995-2000 | 2.32 (2.16 , 2.47) | 2000-2006 | -0.2 (-0.29 , -0.1) | 2006-2011 | -2.04 (-2.2 , -1.88) | 2011-2021 | 0.22 (0.15 , 0.29) |  |  |
| Age Standardized Mortality rate | | | | | | | | | | | | |
| Armenia | 1992-2001 | -5.34 (-5.85 , -4.82) | 2001-2007 | -2.8 (-4.06 , -1.52) | 2007-2021 | -5.75 (-6.01 , -5.49) | 2005-2011 | -6.85 (-7.32 , -6.37) |  |  |  |  |
| Azerbaijan | 1992-1994 | 0.83 (-1.3 , 3.01) | 1994-1997 | -6.19 (-8.27 , -4.08) | 1997-2005 | -3.83 (-4.13 , -3.53) | 2009-2021 | -4.61 (-4.95 , -4.26) | 2011-2017 | -5.02 (-5.51 , -4.53) | 2017-2021 | -6.58 (-7.28 , -5.87) |
| Georgia | 1992-2000 | -1.29 (-1.91 , -0.67) | 2000-2006 | -7.63 (-8.82 , -6.41) | 2006-2009 | -0.02 (-6.01 , 6.35) | 2014-2021 | -5.89 (-6.62 , -5.16) |  |  |  |  |
| Kazakhstan | 1992-1995 | 4.3 (1.47 , 7.21) | 1995-2006 | -3.76 (-4.16 , -3.35) | 2006-2014 | -8.63 (-9.3 , -7.96) | 2006-2019 | -6.72 (-6.87 , -6.57) |  |  |  |  |
| Kyrgyzstan | 1992-1994 | 1.93 (-0.68 , 4.6) | 1994-1998 | -4.13 (-5.32 , -2.92) | 1998-2006 | -3.14 (-3.48 , -2.79) | 2008-2011 | -3.45 (-6.8 , 0.02) | 2019-2021 | -4.89 (-7.62 , -2.08) |  |  |
| Mongolia | 1992-1996 | -2.71 (-3.91 , -1.51) | 1996-2005 | -4.92 (-5.31 , -4.52) | 2005-2008 | -6.32 (-9.86 , -2.64) | 2009-2013 | -7.75 (-8.89 , -6.6) | 2011-2014 | -8.91 (-12 , -5.71) | 2014-2021 | -6.09 (-6.57 , -5.61) |
| Tajikistan | 1992-1994 | 4.34 (2.04 , 6.7) | 1994-2003 | -4.74 (-4.98 , -4.5) | 2003-2009 | -3.89 (-4.43 , -3.36) | 2001-2006 | -1.28 (-2.26 , -0.29) | 2013-2021 | -5.63 (-5.88 , -5.37) |  |  |
| Turkmenistan | 1992-1994 | 3.4 (0.12 , 6.79) | 1994-1997 | -1.63 (-4.63 , 1.48) | 1997-2001 | -6.32 (-7.79 , -4.82) | 2005-2010 | -5.86 (-6.84 , -4.87) | 2006-2010 | -9.99 (-11.4 , -8.56) | 2010-2021 | -3.63 (-3.86 , -3.41) |
| Uzbekistan | 1992-1995 | 0.71 (-0.81 , 2.25) | 1995-2001 | -4.25 (-4.9 , -3.6) | 2001-2005 | -2.6 (-4.12 , -1.06) | 2010-2015 | -8.05 (-8.51 , -7.59) | 2010-2016 | -4.62 (-5.32 , -3.91) | 2016-2021 | -6.9 (-7.63 , -6.17) |
| Age Standardized DALY rate | | | | | | | | | | | | |
| Armenia | 1992-2000 | -1.56 (-1.72 , -1.41) | 2000-2005 | 2.58 (2.11 , 3.06) | 2005-2015 | -1.24 (-1.38 , -1.11) | 2015-2021 | -0.09 (-0.37 , 0.18) |  |  |  |  |
| Azerbaijan | 1992-1994 | 0.19 (-1.06 , 1.46) | 1994-1997 | -3.38 (-4.56 , -2.18) | 1997-2004 | -1.42 (-1.64 , -1.2) | 2004-2008 | -3.29 (-3.92 , -2.66) | 2008-2014 | -2.44 (-2.74 , -2.13) | 2014-2021 | -0.93 (-1.12 , -0.73) |
| Georgia | 1992-2009 | -0.56 (-0.62 , -0.5) | 2009-2014 | -1.25 (-1.82 , -0.67) | 2014-2021 | -0.59 (-0.84 , -0.34) |  |  |  |  |  |  |
| Kazakhstan | 1992-1995 | 4.17 (3.01 , 5.35) | 1995-1999 | -1.25 (-2.34 , -0.15) | 1999-2004 | 0.23 (-0.49 , 0.96) | 2007-2010 | -1.02 (-2.26 , 0.23) | 2010-2015 | -8.02 (-8.76 , -7.28) | 2015-2021 | -0.9 (-1.39 , -0.42) |
| Kyrgyzstan | 1992-1994 | 1.59 (0.38 , 2.82) | 1994-1999 | -1.73 (-2.1 , -1.37) | 1999-2007 | 0.14 (-0.01 , 0.3) | 2011-2015 | -4.74 (-5.88 , -3.58) | 2010-2015 | -6.36 (-6.75 , -5.98) | 2015-2021 | -0.92 (-1.16 , -0.67) |
| Mongolia | 1992-1996 | -2.03 (-2.96 , -1.09) | 1996-2000 | -4.49 (-5.79 , -3.16) | 2000-2011 | -2.69 (-2.89 , -2.49) | 2006-2011 | -2.77 (-3.11 , -2.43) | 2015-2021 | -1.85 (-2.27 , -1.42) |  |  |
| Tajikistan | 1992-1994 | 6.44 (5.34 , 7.54) | 1994-2001 | -2.67 (-2.84 , -2.49) | 2001-2006 | -0.98 (-1.31 , -0.64) | 2011-2014 | -8.28 (-9.82 , -6.71) | 2011-2014 | -9.96 (-10.92 , -8.99) | 2014-2021 | -1.64 (-1.8 , -1.49) |
| Turkmenistan | 1992-1997 | -0.1 (-0.45 , 0.25) | 1997-2007 | -0.84 (-0.98 , -0.69) | 2007-2011 | -2.46 (-3.29 , -1.63) | 2010-2021 | -2.26 (-2.37 , -2.14) | 2014-2021 | -0.49 (-0.72 , -0.27) |  |  |
| Uzbekistan | 1992-1995 | 0.09 (-0.61 , 0.8) | 1995-2006 | -2.15 (-2.26 , -2.03) | 2006-2010 | -4.2 (-4.97 , -3.43) | 2009-2021 | -1.33 (-1.44 , -1.22) |  |  |  |  |

Abbreviation: APC, annual percent change; CI, confidence interval;

**Table S4.** Age-Period-Cohort Model Analysis of Cystic Echinococcosis Incidence Rates from 1992 to 2021.

|  | **Armenia** | **Azerbaijan** | **Georgia** | **Kazakhstan** | **Kyrgyzstan** | **Mongolia** | **Tajikistan** | **Turkmenistan** | **Uzbekistan** |
| --- | --- | --- | --- | --- | --- | --- | --- | --- | --- |
| **Age** |  |  |  |  |  |  |  |  |  |
| **< 5** | 0.76 (0.51, 1.12) | 1.09 (0.73, 1.64) | 0.88 (0.6, 1.27) | 0.48 (0.36, 0.64) | 0.88 (0.65, 1.2) | 1.19 (0.46, 3.09) | 1.5 (1.11, 2.03) | 1.75 (1.23, 2.48) | 1.02 (0.84, 1.25) |
| **5-9** | 0.77 (0.54, 1.1) | 1.08 (0.75, 1.55) | 0.89 (0.63, 1.26) | 0.52 (0.4, 0.67) | 0.86 (0.65, 1.13) | 1.17 (0.51, 2.68) | 1.4 (1.07, 1.82) | 1.63 (1.2, 2.22) | 1 (0.84, 1.2) |
| **10-14** | 0.79 (0.58, 1.08) | 1.07 (0.77, 1.48) | 0.91 (0.66, 1.25) | 0.56 (0.44, 0.7) | 0.84 (0.66, 1.08) | 1.15 (0.56, 2.35) | 1.3 (1.03, 1.64) | 1.52 (1.16, 1.99) | 0.98 (0.84, 1.16) |
| **15-19** | 0.81 (0.61, 1.07) | 1.06 (0.79, 1.41) | 0.93 (0.69, 1.24) | 0.6 (0.49, 0.73) | 0.82 (0.66, 1.03) | 1.13 (0.61, 2.08) | 1.21 (0.99, 1.49) | 1.42 (1.11, 1.8) | 0.96 (0.83, 1.11) |
| **20-24** | 0.82 (0.64, 1.06) | 1.05 (0.81, 1.36) | 0.94 (0.72, 1.24) | 0.64 (0.53, 0.78) | 0.8 (0.65, 0.98) | 1.11 (0.65, 1.88) | 1.13 (0.94, 1.35) | 1.32 (1.07, 1.63) | 0.94 (0.83, 1.08) |
| **25-29** | 0.84 (0.66, 1.07) | 1.03 (0.81, 1.32) | 0.96 (0.74, 1.24) | 0.69 (0.58, 0.82) | 0.78 (0.65, 0.95) | 1.09 (0.67, 1.75) | 1.05 (0.89, 1.24) | 1.23 (1.01, 1.5) | 0.93 (0.82, 1.05) |
| **30-34** | 0.86 (0.69, 1.08) | 1.02 (0.81, 1.29) | 0.98 (0.76, 1.25) | 0.74 (0.62, 0.88) | 0.77 (0.63, 0.92) | 1.07 (0.67, 1.7) | 0.98 (0.83, 1.15) | 1.15 (0.95, 1.39) | 0.91 (0.8, 1.02) |
| **35-39** | 0.88 (0.7, 1.11) | 1.01 (0.8, 1.29) | 0.99 (0.78, 1.27) | 0.79 (0.67, 0.95) | 0.75 (0.62, 0.91) | 1.05 (0.63, 1.73) | 0.91 (0.76, 1.09) | 1.07 (0.88, 1.31) | 0.89 (0.78, 1.01) |
| **40-44** | 0.9 (0.71, 1.14) | 1 (0.77, 1.3) | 1.01 (0.79, 1.29) | 0.85 (0.71, 1.02) | 0.73 (0.59, 0.9) | 1.03 (0.58, 1.81) | 0.85 (0.7, 1.03) | 1 (0.8, 1.25) | 0.87 (0.76, 1) |
| **45-49** | 0.92 (0.7, 1.2) | 0.99 (0.74, 1.32) | 1.03 (0.8, 1.33) | 0.92 (0.75, 1.12) | 0.71 (0.57, 0.9) | 1.01 (0.52, 1.95) | 0.79 (0.63, 0.99) | 0.93 (0.73, 1.19) | 0.85 (0.74, 0.99) |
| **50-54** | 0.94 (0.7, 1.26) | 0.98 (0.71, 1.36) | 1.05 (0.8, 1.37) | 0.98 (0.79, 1.22) | 0.7 (0.54, 0.9) | 0.99 (0.46, 2.14) | 0.74 (0.57, 0.95) | 0.87 (0.65, 1.15) | 0.84 (0.71, 0.99) |
| **55-59** | 0.96 (0.69, 1.33) | 0.97 (0.67, 1.4) | 1.07 (0.8, 1.42) | 1.06 (0.83, 1.35) | 0.68 (0.51, 0.91) | 0.97 (0.4, 2.36) | 0.68 (0.51, 0.91) | 0.81 (0.59, 1.12) | 0.82 (0.68, 0.99) |
| **60-64** | 0.98 (0.68, 1.41) | 0.96 (0.64, 1.44) | 1.09 (0.8, 1.48) | 1.13 (0.87, 1.48) | 0.67 (0.49, 0.91) | 0.95 (0.34, 2.63) | 0.64 (0.46, 0.88) | 0.75 (0.52, 1.09) | 0.8 (0.65, 0.99) |
| **65-69** | 1 (0.67, 1.5) | 0.95 (0.6, 1.5) | 1.11 (0.79, 1.54) | 1.22 (0.9, 1.64) | 0.65 (0.46, 0.92) | 0.93 (0.3, 2.94) | 0.59 (0.41, 0.86) | 0.7 (0.47, 1.06) | 0.79 (0.63, 0.99) |
| **70-74** | 1.02 (0.65, 1.6) | 0.94 (0.57, 1.56) | 1.13 (0.79, 1.62) | 1.31 (0.94, 1.81) | 0.64 (0.43, 0.94) | 0.92 (0.25, 3.29) | 0.55 (0.37, 0.83) | 0.66 (0.42, 1.03) | 0.77 (0.6, 1) |
| **75-79** | 1.04 (0.64, 1.71) | 0.93 (0.53, 1.62) | 1.15 (0.78, 1.69) | 1.4 (0.98, 2.01) | 0.62 (0.41, 0.95) | 0.9 (0.22, 3.7) | 0.51 (0.33, 0.8) | 0.61 (0.37, 1.01) | 0.76 (0.57, 1) |
| **80-84** | 1.07 (0.62, 1.83) | 0.92 (0.5, 1.68) | 1.17 (0.77, 1.78) | 1.51 (1.02, 2.23) | 0.61 (0.38, 0.96) | 0.88 (0.19, 4.16) | 0.48 (0.29, 0.78) | 0.57 (0.33, 0.98) | 0.74 (0.55, 1.01) |
| **85-90** | 1.09 (0.61, 1.96) | 0.91 (0.47, 1.75) | 1.19 (0.76, 1.87) | 1.62 (1.06, 2.47) | 0.59 (0.36, 0.98) | 0.87 (0.16, 4.69) | 0.45 (0.26, 0.76) | 0.53 (0.29, 0.96) | 0.73 (0.52, 1.01) |
| **90-94** | 1.11 (0.59, 2.1) | 0.9 (0.44, 1.83) | 1.21 (0.74, 1.97) | 1.74 (1.1, 2.74) | 0.58 (0.34, 0.99) | 0.85 (0.14, 5.29) | 0.42 (0.23, 0.74) | 0.5 (0.26, 0.94) | 0.71 (0.5, 1.02) |
| **95 +** | 1.14 (0.58, 2.25) | 0.89 (0.41, 1.91) | 1.23 (0.73, 2.08) | 1.87 (1.15, 3.04) | 0.57 (0.32, 1.01) | 0.83 (0.12, 5.97) | 0.39 (0.21, 0.72) | 0.46 (0.23, 0.92) | 0.7 (0.48, 1.02) |
| **Period** |  |  |  |  |  |  |  |  |  |
| **1992-1996** | 0.89 (0.74, 1.07) | 1 (0.83, 1.22) | 0.92 (0.77, 1.1) | 0.75 (0.66, 0.86) | 0.9 (0.78, 1.04) | 1.04 (0.69, 1.56) | 0.88 (0.77, 1) | 0.99 (0.85, 1.16) | 0.95 (0.86, 1.04) |
| **1997-2001** | 0.89 (0.75, 1.05) | 1 (0.84, 1.19) | 0.92 (0.78, 1.1) | 0.85 (0.75, 0.96) | 0.91 (0.8, 1.03) | 1.01 (0.72, 1.43) | 0.91 (0.82, 1.02) | 1 (0.87, 1.14) | 1 (0.92, 1.09) |
| **2002-2006** | 1 (1, 1) | 1 (1, 1) | 1 (1, 1) | 1 (1, 1) | 1 (1, 1) | 1 (1, 1) | 1 (1, 1) | 1 (1, 1) | 1 (1, 1) |
| **2007-2011** | 1.01 (0.85, 1.18) | 0.98 (0.83, 1.15) | 1.01 (0.84, 1.2) | 1.37 (1.23, 1.54) | 1.08 (0.96, 1.22) | 0.97 (0.7, 1.35) | 0.98 (0.88, 1.09) | 0.97 (0.85, 1.11) | 0.92 (0.85, 1) |
| **2012-2016** | 0.97 (0.81, 1.17) | 0.95 (0.79, 1.15) | 0.99 (0.82, 1.19) | 1.09 (0.95, 1.24) | 0.85 (0.74, 0.98) | 0.94 (0.62, 1.41) | 0.69 (0.61, 0.79) | 0.76 (0.65, 0.89) | 0.89 (0.81, 0.98) |
| **2017-2021** | 0.98 (0.8, 1.21) | 0.96 (0.77, 1.2) | 1 (0.82, 1.22) | 1 (0.86, 1.16) | 0.78 (0.66, 0.92) | 0.96 (0.57, 1.59) | 0.63 (0.54, 0.74) | 0.72 (0.6, 0.87) | 0.89 (0.8, 0.99) |
| **Cohort** |  |  |  |  |  |  |  |  |  |
| **1897-1901** | 0.87 (0, 9830.63) | 1.17 (0, 50424.46) | 0.73 (0, 125.13) | 0.66 (0, 179.65) | 3.92 (0.01, 3090.12) | 1.2 (0,1400557363232.44) | 3.53 (0, 6174.39) | 1.91 (0, 16450.17) | 1.77 (0.02, 161.37) |
| **1902-1906** | 0.88 (0.02, 49.07) | 1.15 (0.02, 60.07) | 0.75 (0.08, 7.44) | 0.64 (0.05, 7.83) | 3.23 (0.19, 55.07) | 1.2 (0, 283986.62) | 3.15 (0.13, 78.75) | 1.9 (0.03, 105.56) | 1.67 (0.27, 10.5) |
| **1907-1911** | 0.89 (0.11, 7.59) | 1.13 (0.13, 9.88) | 0.77 (0.19, 3.08) | 0.6 (0.14, 2.61) | 2.66 (0.52, 13.64) | 1.18 (0, 566.45) | 2.75 (0.41, 18.46) | 1.87 (0.19, 18.5) | 1.58 (0.54, 4.64) |
| **1912-1916** | 0.89 (0.23, 3.45) | 1.1 (0.26, 4.72) | 0.81 (0.31, 2.11) | 0.57 (0.22, 1.52) | 2.2 (0.74, 6.5) | 1.15 (0.03, 44.53) | 2.37 (0.68, 8.28) | 1.81 (0.44, 7.39) | 1.48 (0.69, 3.18) |
| **1917-1921** | 0.88 (0.32, 2.43) | 1.08 (0.34, 3.38) | 0.83 (0.39, 1.77) | 0.58 (0.28, 1.23) | 1.86 (0.8, 4.33) | 1.12 (0.08, 15.26) | 2.06 (0.82, 5.18) | 1.74 (0.64, 4.76) | 1.39 (0.76, 2.55) |
| **1922-1926** | 0.87 (0.42, 1.78) | 1.06 (0.44, 2.52) | 0.81 (0.45, 1.45) | 0.61 (0.35, 1.04) | 1.6 (0.86, 2.99) | 1.1 (0.18, 6.94) | 1.81 (0.94, 3.49) | 1.65 (0.8, 3.4) | 1.31 (0.84, 2.04) |
| **1927-1931** | 0.87 (0.55, 1.38) | 1.05 (0.58, 1.89) | 0.76 (0.49, 1.17) | 0.64 (0.44, 0.94) | 1.41 (0.9, 2.22) | 1.1 (0.27, 4.41) | 1.64 (1.03, 2.61) | 1.56 (0.94, 2.59) | 1.25 (0.91, 1.7) |
| **1932-1936** | 0.91 (0.63, 1.31) | 1.05 (0.67, 1.63) | 0.76 (0.53, 1.1) | 0.68 (0.5, 0.94) | 1.27 (0.87, 1.83) | 1.09 (0.38, 3.11) | 1.48 (1.03, 2.12) | 1.48 (0.99, 2.2) | 1.19 (0.93, 1.52) |
| **1937-1941** | 0.94 (0.69, 1.29) | 1.04 (0.72, 1.48) | 0.82 (0.6, 1.12) | 0.73 (0.57, 0.94) | 1.18 (0.87, 1.61) | 1.07 (0.45, 2.52) | 1.36 (1.01, 1.83) | 1.38 (0.99, 1.92) | 1.15 (0.93, 1.4) |
| **1942-1946** | 0.97 (0.71, 1.32) | 1.03 (0.73, 1.46) | 0.89 (0.66, 1.19) | 0.77 (0.62, 0.97) | 1.12 (0.84, 1.49) | 1.05 (0.51, 2.17) | 1.25 (0.96, 1.64) | 1.28 (0.95, 1.71) | 1.1 (0.92, 1.32) |
| **1947-1951** | 0.99 (0.75, 1.3) | 1.02 (0.74, 1.41) | 0.94 (0.72, 1.24) | 0.85 (0.69, 1.05) | 1.09 (0.84, 1.41) | 1.04 (0.54, 1.98) | 1.15 (0.9, 1.47) | 1.18 (0.9, 1.54) | 1.06 (0.9, 1.26) |
| **1952-1956** | 1 (0.79, 1.26) | 1.01 (0.78, 1.31) | 0.98 (0.77, 1.25) | 0.93 (0.78, 1.11) | 1.05 (0.85, 1.3) | 1.02 (0.58, 1.8) | 1.07 (0.87, 1.3) | 1.08 (0.87, 1.35) | 1.03 (0.9, 1.18) |
| **1957-1961** | 1 (1, 1) | 1 (1, 1) | 1 (1, 1) | 1 (1, 1) | 1 (1, 1) | 1 (1, 1) | 1 (1, 1) | 1 (1, 1) | 1 (1, 1) |
| **1962-1966** | 1.01 (0.81, 1.25) | 0.99 (0.79, 1.24) | 1.02 (0.8, 1.29) | 1.08 (0.92, 1.27) | 0.99 (0.82, 1.19) | 0.98 (0.61, 1.57) | 0.94 (0.8, 1.12) | 0.93 (0.76, 1.13) | 0.99 (0.87, 1.12) |
| **1967-1971** | 1.04 (0.82, 1.32) | 0.98 (0.77, 1.24) | 1.05 (0.81, 1.36) | 1.2 (1.01, 1.42) | 1 (0.82, 1.22) | 0.96 (0.6, 1.56) | 0.9 (0.75, 1.07) | 0.87 (0.71, 1.06) | 1 (0.88, 1.13) |
| **1972-1976** | 1.08 (0.83, 1.4) | 0.97 (0.75, 1.25) | 1.08 (0.82, 1.43) | 1.32 (1.1, 1.59) | 1.03 (0.84, 1.27) | 0.95 (0.58, 1.56) | 0.86 (0.72, 1.03) | 0.81 (0.66, 1.01) | 1.01 (0.89, 1.16) |
| **1977-1981** | 1.1 (0.84, 1.44) | 0.96 (0.73, 1.25) | 1.1 (0.81, 1.48) | 1.44 (1.18, 1.75) | 1.1 (0.88, 1.36) | 0.93 (0.55, 1.57) | 0.82 (0.68, 1) | 0.76 (0.61, 0.95) | 1.02 (0.89, 1.17) |
| **1982-1986** | 1.13 (0.85, 1.49) | 0.95 (0.72, 1.25) | 1.09 (0.79, 1.51) | 1.57 (1.28, 1.94) | 1.19 (0.95, 1.48) | 0.92 (0.53, 1.58) | 0.78 (0.65, 0.95) | 0.71 (0.56, 0.9) | 1.03 (0.89, 1.19) |
| **1987-1991** | 1.18 (0.88, 1.59) | 0.94 (0.7, 1.26) | 1.07 (0.76, 1.51) | 1.74 (1.4, 2.16) | 1.27 (1.01, 1.6) | 0.91 (0.52, 1.6) | 0.75 (0.62, 0.92) | 0.66 (0.52, 0.85) | 1.04 (0.9, 1.21) |
| **1992-1996** | 1.24 (0.9, 1.71) | 0.93 (0.68, 1.27) | 1.03 (0.7, 1.52) | 1.87 (1.49, 2.36) | 1.38 (1.09, 1.75) | 0.9 (0.5, 1.63) | 0.72 (0.58, 0.89) | 0.61 (0.48, 0.79) | 1.04 (0.89, 1.21) |
| **1997-2001** | 1.28 (0.89, 1.86) | 0.92 (0.65, 1.3) | 1.01 (0.64, 1.57) | 1.98 (1.53, 2.56) | 1.51 (1.17, 1.94) | 0.87 (0.46, 1.67) | 0.67 (0.54, 0.84) | 0.57 (0.43, 0.75) | 1.04 (0.88, 1.23) |
| **2002-2006** | 1.33 (0.86, 2.04) | 0.91 (0.61, 1.35) | 1 (0.59, 1.69) | 2.09 (1.57, 2.79) | 1.5 (1.13, 1.99) | 0.84 (0.41, 1.74) | 0.63 (0.49, 0.8) | 0.52 (0.39, 0.71) | 1.05 (0.87, 1.26) |
| **2007-2011** | 1.34 (0.8, 2.24) | 0.89 (0.56, 1.42) | 0.99 (0.52, 1.88) | 2.07 (1.49, 2.88) | 1.26 (0.9, 1.76) | 0.8 (0.35, 1.82) | 0.58 (0.44, 0.76) | 0.47 (0.33, 0.68) | 1.01 (0.81, 1.25) |
| **2012-2016** | 1.29 (0.59, 2.8) | 0.86 (0.44, 1.66) | 0.98 (0.39, 2.47) | 1.93 (1.23, 3.03) | 1.05 (0.66, 1.65) | 0.71 (0.24, 2.06) | 0.54 (0.38, 0.76) | 0.42 (0.26, 0.7) | 0.9 (0.68, 1.2) |
| **2017-2021** | 1.25 (0.12, 13) | 0.8 (0.11, 5.75) | 0.99 (0.07, 13.46) | 1.93 (0.56, 6.64) | 1.09 (0.35, 3.39) | 0.51 (0.03, 7.85) | 0.5 (0.23, 1.09) | 0.42 (0.12, 1.55) | 0.72 (0.36, 1.45) |

**Table S5.** Age-Period-Cohort Model Analysis of Cystic Echinococcosis Mortality Rates from 1992 to 2021.

|  | **Armenia** | **Azerbaijan** | **Georgia** | **Kazakhstan** | **Kyrgyzstan** | **Mongolia** | **Tajikistan** | **Turkmenistan** | **Uzbekistan** |
| --- | --- | --- | --- | --- | --- | --- | --- | --- | --- |
| **Age** |  |  |  |  |  |  |  |  |  |
| **< 5** | 11.31 (0, 3932255.67) | 14.52 (0.03, 7473.64) | 8.46 (0, 268218.11) | 12.42 (0.22, 710.02) | 9.59 (0.14, 678.31) | 9.02 (0, 42295.85) | 10.62 (0.22, 504.73) | 6.26 (0, 12677.53) | 7.24 (0.84, 62.2) |
| **5-9** | 8.73 (0, 1320791.06) | 10.9 (0.03, 3797.76) | 6.76 (0, 109244.78) | 9.2 (0.21, 402.18) | 7.38 (0.14, 399.07) | 6.94 (0, 19032.87) | 8.19 (0.22, 310) | 5 (0, 6298.55) | 5.77 (0.77, 43.5) |
| **10-14** | 6.73 (0, 455775.74) | 8.18 (0.03, 1969.04) | 5.41 (0, 45486.27) | 6.82 (0.2, 230.78) | 5.68 (0.14, 238.5) | 5.35 (0, 8901.08) | 6.32 (0.21, 193.59) | 3.99 (0, 3232.25) | 4.6 (0.69, 30.65) |
| **15-19** | 5.19 (0, 162602.49) | 6.14 (0.04, 1046.18) | 4.33 (0, 19460.54) | 5.06 (0.19, 134.56) | 4.37 (0.13, 145.29) | 4.12 (0, 4362.23) | 4.88 (0.19, 123.31) | 3.19 (0.01, 1725.03) | 3.66 (0.62, 21.8) |
| **20-24** | 4.01 (0, 60456.07) | 4.61 (0.04, 572.61) | 3.46 (0, 8611.06) | 3.75 (0.18, 80.02) | 3.37 (0.13, 90.58) | 3.17 (0, 2262.04) | 3.76 (0.18, 80.41) | 2.55 (0.01, 965.14) | 2.92 (0.54, 15.68) |
| **25-29** | 3.09 (0, 23663.72) | 3.46 (0.04, 324.88) | 2.77 (0, 3973.38) | 2.78 (0.16, 48.76) | 2.59 (0.12, 58.08) | 2.44 (0, 1254.53) | 2.9 (0.16, 53.91) | 2.04 (0.01, 571.19) | 2.33 (0.47, 11.42) |
| **30-34** | 2.38 (0, 9874.55) | 2.6 (0.04, 192.41) | 2.21 (0, 1931.54) | 2.06 (0.14, 30.6) | 1.99 (0.1, 38.49) | 1.88 (0, 752.41) | 2.24 (0.13, 37.31) | 1.63 (0.01, 360.95) | 1.85 (0.41, 8.46) |
| **35-39** | 1.84 (0, 4459.87) | 1.95 (0.03, 119.84) | 1.77 (0, 1001.45) | 1.53 (0.12, 19.9) | 1.54 (0.09, 26.5) | 1.45 (0, 492.94) | 1.73 (0.11, 26.75) | 1.3 (0.01, 245.7) | 1.48 (0.34, 6.37) |
| **40-44** | 1.42 (0, 2218.25) | 1.46 (0.03, 79.05) | 1.42 (0, 561.6) | 1.13 (0.1, 13.48) | 1.18 (0.07, 19.05) | 1.11 (0, 355.4) | 1.33 (0.09, 19.93) | 1.04 (0.01, 181.41) | 1.18 (0.28, 4.9) |
| **45-49** | 1.09 (0, 1237.18) | 1.1 (0.02, 55.54) | 1.13 (0, 345.65) | 0.84 (0.07, 9.56) | 0.91 (0.06, 14.34) | 0.86 (0, 282.92) | 1.03 (0.07, 15.46) | 0.83 (0, 145.84) | 0.94 (0.23, 3.85) |
| **50-54** | 0.84 (0, 786.47) | 0.82 (0.02, 41.7) | 0.91 (0, 236.58) | 0.62 (0.05, 7.11) | 0.7 (0.04, 11.32) | 0.66 (0, 248.26) | 0.79 (0.05, 12.47) | 0.66 (0, 127.61) | 0.75 (0.18, 3.09) |
| **55-59** | 0.65 (0, 576.25) | 0.62 (0.01, 33.45) | 0.72 (0, 181.7) | 0.46 (0.04, 5.55) | 0.54 (0.03, 9.35) | 0.51 (0, 238.63) | 0.61 (0.04, 10.45) | 0.53 (0, 121.01) | 0.6 (0.14, 2.54) |
| **60-64** | 0.5 (0, 488.22) | 0.46 (0.01, 28.59) | 0.58 (0, 156.99) | 0.34 (0.03, 4.53) | 0.42 (0.02, 8.06) | 0.39 (0, 248.93) | 0.47 (0.02, 9.07) | 0.42 (0, 123.48) | 0.47 (0.11, 2.13) |
| **65-69** | 0.39 (0, 475.56) | 0.35 (0, 25.87) | 0.46 (0, 151.86) | 0.25 (0.02, 3.85) | 0.32 (0.01, 7.23) | 0.3 (0, 278.74) | 0.36 (0.02, 8.11) | 0.34 (0, 134.35) | 0.38 (0.08, 1.82) |
| **70-74** | 0.3 (0, 525.64) | 0.26 (0, 24.62) | 0.37 (0, 162.74) | 0.19 (0.01, 3.38) | 0.25 (0.01, 6.69) | 0.23 (0, 331.41) | 0.28 (0.01, 7.45) | 0.27 (0, 154.43) | 0.3 (0.06, 1.58) |
| **75-79** | 0.23 (0, 648.1) | 0.2 (0, 24.46) | 0.3 (0, 190.54) | 0.14 (0.01, 3.06) | 0.19 (0.01, 6.37) | 0.18 (0, 414.16) | 0.22 (0.01, 7) | 0.22 (0, 185.88) | 0.24 (0.04, 1.39) |
| **80-84** | 0.18 (0, 875.38) | 0.15 (0, 25.18) | 0.24 (0, 240.22) | 0.1 (0, 2.84) | 0.15 (0, 6.2) | 0.14 (0, 539.29) | 0.17 (0, 6.7) | 0.17 (0, 232.42) | 0.19 (0.03, 1.24) |
| **85-90** | 0.14 (0, 1273.75) | 0.11 (0, 26.71) | 0.19 (0, 321.68) | 0.08 (0, 2.68) | 0.11 (0, 6.15) | 0.11 (0, 726.36) | 0.13 (0, 6.52) | 0.14 (0, 299.93) | 0.15 (0.02, 1.11) |
| **90-94** | 0.11 (0, 1968.09) | 0.08 (0, 29.03) | 0.15 (0, 452.26) | 0.06 (0, 2.57) | 0.09 (0, 6.2) | 0.08 (0, 1005.85) | 0.1 (0, 6.43) | 0.11 (0, 397.24) | 0.12 (0.01, 1.01) |
| **95 +** | 0.08 (0, 3191.14) | 0.06 (0, 32.19) | 0.12 (0, 661.23) | 0.04 (0, 2.5) | 0.07 (0, 6.33) | 0.06 (0, 1425.12) | 0.08 (0, 6.4) | 0.09 (0, 537.52) | 0.1 (0.01, 0.92) |
| **Period** |  |  |  |  |  |  |  |  |  |
| **1992-1996** | 1.71 (0.02, 124.1) | 1.66 (0.14, 19.06) | 1.49 (0.05, 44.67) | 1.33 (0.3, 5.98) | 1.41 (0.25, 8.02) | 1.63 (0.04, 65.97) | 1.63 (0.32, 8.26) | 1.34 (0.05, 38.68) | 1.44 (0.58, 3.58) |
| **1997-2001** | 1.24 (0.02, 100.81) | 1.27 (0.11, 15.15) | 1.37 (0.05, 38.85) | 1.19 (0.26, 5.42) | 1.19 (0.21, 6.93) | 1.27 (0.03, 54.38) | 1.33 (0.26, 6.7) | 1.21 (0.04, 34.37) | 1.22 (0.49, 3.02) |
| **2002-2006** | 1 (1, 1) | 1 (1, 1) | 1 (1, 1) | 1 (1, 1) | 1 (1, 1) | 1 (1, 1) | 1 (1, 1) | 1 (1, 1) | 1 (1, 1) |
| **2007-2011** | 0.81 (0.01, 88.43) | 0.71 (0.05, 11.11) | 0.79 (0.02, 35.45) | 0.7 (0.13, 3.79) | 0.76 (0.11, 5.12) | 0.79 (0.01, 43.72) | 0.78 (0.13, 4.62) | 0.74 (0.02, 28.15) | 0.77 (0.29, 2.04) |
| **2012-2016** | 0.61 (0, 100.74) | 0.53 (0.03, 10.19) | 0.66 (0.01, 40.1) | 0.44 (0.07, 2.98) | 0.54 (0.07, 4.25) | 0.59 (0.01, 42.5) | 0.59 (0.09, 3.91) | 0.56 (0.01, 26.85) | 0.61 (0.22, 1.7) |
| **2017-2021** | 0.44 (0, 124.67) | 0.4 (0.02, 9.51) | 0.51 (0.01, 48.5) | 0.32 (0.04, 2.57) | 0.39 (0.04, 3.64) | 0.43 (0, 43.43) | 0.46 (0.06, 3.44) | 0.47 (0.01, 25.74) | 0.47 (0.16, 1.38) |
| **Cohort** |  |  |  |  |  |  |  |  |  |
| **1897-1901** | 12.51 (0, 2599219379128390000) | 27.83 (0, 247617684930.32) | 12.69 (0, 3638425346636.78) | 27.37 (0, 175281895.03) | 20.89 (0, 551378002.17) | 17.59 (0, 8.13605737707431E+27) | 14.1 (0, 402889611.96) | 26.5 (0, 41860206391059000000) | 15.13 (0, 136846.79) |
| **1902-1906** | 11.3 (0, 25648018406.45) | 15.15 (0, 9451750.51) | 8.79 (0, 44562472.53) | 21.62 (0, 193721.19) | 15.77 (0, 344988.88) | 16.3 (0, 375343243297776) | 11.21 (0, 179621.64) | 17.55 (0, 437927070526.47) | 10.53 (0.05, 2025.27) |
| **1907-1911** | 10.02 (0, 32112621.74) | 10.27 (0, 185095.22) | 6.8 (0, 631778.09) | 17.59 (0.03, 11840.94) | 11.76 (0.01, 16791.44) | 14.23 (0, 2150742129.36) | 9.58 (0.01, 12902.37) | 12.56 (0, 347393964.65) | 8.1 (0.18, 364.63) |
| **1912-1916** | 8.97 (0, 1597000.94) | 7.92 (0, 24103.29) | 5.59 (0, 67115.58) | 14.61 (0.09, 2454.39) | 9.02 (0.02, 3292.62) | 11.78 (0, 13211778.48) | 8.24 (0.02, 2828.01) | 8.96 (0, 3483012.06) | 6.82 (0.31, 151.34) |
| **1917-1921** | 6.65 (0, 442885.33) | 6.41 (0, 8770.47) | 4.46 (0, 19683.74) | 11.16 (0.12, 1041.98) | 7.2 (0.04, 1368.54) | 9.7 (0, 1146911.95) | 6.97 (0.04, 1191.16) | 6.62 (0, 357821.11) | 5.79 (0.38, 88.45) |
| **1922-1926** | 5.71 (0, 100568.83) | 5.88 (0.01, 3506.53) | 3.73 (0, 8100.8) | 8.44 (0.15, 460.47) | 6.15 (0.06, 593.67) | 7.84 (0, 182694.52) | 5.87 (0.06, 551.03) | 5.15 (0, 55687.8) | 5.14 (0.49, 53.69) |
| **1927-1931** | 4.46 (0, 22547.75) | 5.35 (0.02, 1322.79) | 3.21 (0, 3417.99) | 6.38 (0.2, 205.72) | 4.98 (0.09, 277.26) | 6.13 (0, 51627.78) | 4.92 (0.08, 290.78) | 4.07 (0, 12042.97) | 4.32 (0.56, 33.41) |
| **1932-1936** | 3.48 (0, 10895.01) | 4.47 (0.03, 650.94) | 2.76 (0, 2013.21) | 4.8 (0.19, 122.57) | 4.05 (0.1, 165.35) | 4.58 (0, 13817.61) | 4.24 (0.1, 179.25) | 3.18 (0, 4239.05) | 3.54 (0.55, 22.9) |
| **1937-1941** | 2.66 (0, 5723.58) | 3.29 (0.03, 338.54) | 2.16 (0, 1144.99) | 3.43 (0.19, 62.84) | 3.02 (0.1, 95.81) | 3.22 (0, 5104.66) | 3.27 (0.1, 110.46) | 2.48 (0, 1859.47) | 2.77 (0.49, 15.79) |
| **1942-1946** | 2.17 (0, 5687.48) | 2.45 (0.02, 293.27) | 1.79 (0, 913.21) | 2.46 (0.14, 41.98) | 2.3 (0.07, 71.51) | 2.38 (0, 2369.4) | 2.5 (0.07, 85.14) | 1.95 (0, 1246.29) | 2.07 (0.37, 11.64) |
| **1947-1951** | 1.47 (0, 4525.93) | 1.76 (0.01, 252.43) | 1.34 (0, 811.26) | 1.82 (0.11, 29.36) | 1.72 (0.06, 51.4) | 1.72 (0, 1514.29) | 1.82 (0.05, 64.97) | 1.54 (0, 914.69) | 1.73 (0.3, 9.78) |
| **1952-1956** | 1.23 (0, 1869.87) | 1.35 (0.02, 116.13) | 1.17 (0, 447.09) | 1.34 (0.11, 16.29) | 1.33 (0.07, 26.24) | 1.32 (0, 790.09) | 1.36 (0.05, 34.76) | 1.25 (0, 364.05) | 1.31 (0.27, 6.29) |
| **1957-1961** | 1 (1, 1) | 1 (1, 1) | 1 (1, 1) | 1 (1, 1) | 1 (1, 1) | 1 (1, 1) | 1 (1, 1) | 1 (1, 1) | 1 (1, 1) |
| **1962-1966** | 0.85 (0, 1571) | 0.76 (0.01, 54.31) | 0.87 (0, 392.6) | 0.75 (0.06, 9.08) | 0.79 (0.04, 14.04) | 0.82 (0, 298.94) | 0.75 (0.04, 15.96) | 0.82 (0, 190.96) | 0.78 (0.17, 3.69) |
| **1967-1971** | 0.69 (0, 5056.67) | 0.58 (0.01, 67.2) | 0.75 (0, 762.93) | 0.57 (0.04, 9.25) | 0.62 (0.03, 14.64) | 0.69 (0, 360.61) | 0.59 (0.02, 15.54) | 0.69 (0, 256.56) | 0.64 (0.12, 3.48) |
| **1972-1976** | 0.52 (0, 16973.63) | 0.45 (0, 96.73) | 0.62 (0, 1604.41) | 0.48 (0.02, 9.97) | 0.52 (0.02, 16.31) | 0.58 (0, 495.67) | 0.47 (0.01, 15.73) | 0.59 (0, 351.01) | 0.54 (0.08, 3.44) |
| **1977-1981** | 0.35 (0, 50364.58) | 0.31 (0, 125.58) | 0.47 (0, 3968.76) | 0.38 (0.01, 11.08) | 0.41 (0.01, 18.97) | 0.47 (0, 796.02) | 0.36 (0.01, 15.43) | 0.51 (0, 523.76) | 0.44 (0.06, 3.28) |
| **1982-1986** | 0.23 (0, 149357.49) | 0.18 (0, 126.58) | 0.35 (0, 11441.46) | 0.26 (0.01, 12.38) | 0.29 (0, 21.46) | 0.37 (0, 1428.03) | 0.26 (0.01, 13.7) | 0.42 (0, 781.55) | 0.34 (0.04, 2.98) |
| **1987-1991** | 0.18 (0, 453323.81) | 0.11 (0, 118.42) | 0.28 (0, 37164.9) | 0.17 (0, 14.17) | 0.22 (0, 23.86) | 0.29 (0, 2512.12) | 0.2 (0, 11.74) | 0.34 (0, 1066.23) | 0.26 (0.02, 2.63) |
| **1992-1996** | 0.13 (0, 1572180.81) | 0.11 (0, 161.37) | 0.2 (0, 132323.19) | 0.12 (0, 20.86) | 0.16 (0, 28.25) | 0.24 (0, 4109.57) | 0.17 (0, 11.77) | 0.29 (0, 1537.8) | 0.22 (0.02, 2.61) |
| **1997-2001** | 0.11 (0, 15452323.56) | 0.09 (0, 258.74) | 0.14 (0, 951601.44) | 0.09 (0, 40.03) | 0.12 (0, 37.98) | 0.16 (0, 6335.88) | 0.12 (0, 11.15) | 0.21 (0, 2718.9) | 0.17 (0.01, 2.47) |
| **2002-2006** | 0.08 (0, 209758624.03) | 0.06 (0, 423.91) | 0.1 (0, 13789178.7) | 0.06 (0, 81.13) | 0.09 (0, 50.91) | 0.12 (0, 9031.65) | 0.08 (0, 9.73) | 0.15 (0, 6211.58) | 0.13 (0.01, 2.33) |
| **2007-2011** | 0.06 (0, 1304840565.08) | 0.04 (0, 604.74) | 0.06 (0, 858493177.28) | 0.04 (0, 120.95) | 0.06 (0, 59.26) | 0.07 (0, 12451.76) | 0.06 (0, 7.85) | 0.1 (0, 18143.37) | 0.1 (0, 2.07) |
| **2012-2016** | 0.04 (0, 54478773190.58) | 0.03 (0, 975.98) | 0.03 (0, 309741753669.04) | 0.03 (0, 330.84) | 0.04 (0, 87.2) | 0.03 (0, 39269.3) | 0.05 (0, 6.92) | 0.07 (0, 108875.78) | 0.07 (0, 1.84) |
| **2017-2021** | 0.03 (0, 10708468446452100) | 0.02 (0, 22518.96) | 0.02 (0, 4471285972823520000) | 0.02 (0, 35108.24) | 0.03 (0, 698.55) | 0.01 (0, 6869599.44) | 0.03 (0, 8.39) | 0.04 (0, 121405526.81) | 0.04 (0, 2.57) |

**Table S6.** Age-Period-Cohort Model Analysis of Cystic Echinococcosis DALY Rates from 1992 to 2021.

|  | **Armenia** | **Azerbaijan** | **Georgia** | **Kazakhstan** | **Kyrgyzstan** | **Mongolia** | **Tajikistan** | **Turkmenistan** | **Uzbekistan** |
| --- | --- | --- | --- | --- | --- | --- | --- | --- | --- |
| **Age** |  |  |  |  |  |  |  |  |  |
| **< 5** | 1.15 (0.62, 2.12) | 2.45 (1.5, 3.99) | 1.52 (0.84, 2.75) | 1.75 (1.25, 2.47) | 2 (1.37, 2.92) | 3.33 (1.31, 8.5) | 3.13 (2.21, 4.45) | 2.4 (1.41, 4.1) | 2.62 (2.1, 3.26) |
| **5-9** | 1.12 (0.64, 1.95) | 2.2 (1.41, 3.45) | 1.46 (0.84, 2.53) | 1.62 (1.18, 2.21) | 1.8 (1.27, 2.54) | 2.89 (1.25, 6.7) | 2.73 (1.99, 3.75) | 2.16 (1.34, 3.47) | 2.32 (1.9, 2.84) |
| **10-14** | 1.09 (0.65, 1.8) | 1.99 (1.32, 2.99) | 1.4 (0.84, 2.34) | 1.49 (1.12, 1.99) | 1.62 (1.18, 2.22) | 2.51 (1.18, 5.33) | 2.38 (1.78, 3.18) | 1.94 (1.27, 2.96) | 2.06 (1.71, 2.47) |
| **15-19** | 1.06 (0.66, 1.68) | 1.79 (1.23, 2.61) | 1.35 (0.84, 2.16) | 1.38 (1.06, 1.8) | 1.46 (1.09, 1.95) | 2.17 (1.1, 4.29) | 2.07 (1.59, 2.7) | 1.74 (1.19, 2.53) | 1.82 (1.53, 2.16) |
| **20-24** | 1.03 (0.67, 1.57) | 1.61 (1.14, 2.29) | 1.29 (0.83, 2.01) | 1.27 (0.99, 1.63) | 1.31 (1, 1.72) | 1.88 (1.01, 3.51) | 1.81 (1.41, 2.31) | 1.56 (1.11, 2.19) | 1.61 (1.37, 1.89) |
| **25-29** | 1 (0.67, 1.48) | 1.46 (1.04, 2.03) | 1.24 (0.82, 1.88) | 1.17 (0.93, 1.48) | 1.18 (0.91, 1.53) | 1.63 (0.91, 2.93) | 1.57 (1.25, 1.99) | 1.4 (1.02, 1.92) | 1.43 (1.23, 1.67) |
| **30-34** | 0.97 (0.66, 1.42) | 1.31 (0.95, 1.82) | 1.19 (0.8, 1.78) | 1.08 (0.86, 1.36) | 1.06 (0.82, 1.37) | 1.42 (0.8, 2.51) | 1.37 (1.09, 1.73) | 1.25 (0.92, 1.71) | 1.27 (1.09, 1.47) |
| **35-39** | 0.94 (0.64, 1.38) | 1.18 (0.85, 1.64) | 1.14 (0.78, 1.69) | 1 (0.8, 1.25) | 0.96 (0.74, 1.24) | 1.23 (0.68, 2.21) | 1.19 (0.94, 1.51) | 1.13 (0.82, 1.55) | 1.12 (0.97, 1.31) |
| **40-44** | 0.92 (0.62, 1.36) | 1.07 (0.76, 1.5) | 1.1 (0.75, 1.62) | 0.92 (0.73, 1.17) | 0.86 (0.66, 1.13) | 1.06 (0.57, 2) | 1.04 (0.81, 1.34) | 1.01 (0.71, 1.43) | 0.99 (0.85, 1.16) |
| **45-49** | 0.89 (0.59, 1.35) | 0.96 (0.67, 1.38) | 1.06 (0.71, 1.57) | 0.85 (0.66, 1.09) | 0.77 (0.58, 1.03) | 0.92 (0.46, 1.84) | 0.91 (0.69, 1.19) | 0.91 (0.62, 1.33) | 0.88 (0.75, 1.04) |
| **50-54** | 0.86 (0.55, 1.36) | 0.87 (0.59, 1.28) | 1.01 (0.67, 1.53) | 0.79 (0.6, 1.02) | 0.7 (0.51, 0.95) | 0.8 (0.37, 1.72) | 0.79 (0.59, 1.06) | 0.81 (0.53, 1.25) | 0.78 (0.65, 0.93) |
| **55-59** | 0.84 (0.51, 1.38) | 0.78 (0.51, 1.2) | 0.97 (0.63, 1.51) | 0.72 (0.54, 0.97) | 0.63 (0.45, 0.88) | 0.69 (0.29, 1.63) | 0.69 (0.5, 0.95) | 0.73 (0.45, 1.19) | 0.69 (0.57, 0.84) |
| **60-64** | 0.82 (0.47, 1.41) | 0.7 (0.44, 1.12) | 0.94 (0.59, 1.49) | 0.67 (0.49, 0.91) | 0.56 (0.39, 0.82) | 0.6 (0.23, 1.56) | 0.6 (0.42, 0.86) | 0.65 (0.38, 1.13) | 0.61 (0.5, 0.76) |
| **65-69** | 0.79 (0.44, 1.44) | 0.63 (0.38, 1.06) | 0.9 (0.54, 1.48) | 0.62 (0.44, 0.87) | 0.51 (0.34, 0.76) | 0.52 (0.18, 1.5) | 0.52 (0.35, 0.77) | 0.59 (0.32, 1.08) | 0.54 (0.43, 0.68) |
| **70-74** | 0.77 (0.4, 1.49) | 0.57 (0.33, 1) | 0.86 (0.5, 1.48) | 0.57 (0.39, 0.83) | 0.46 (0.29, 0.71) | 0.45 (0.14, 1.44) | 0.46 (0.3, 0.7) | 0.53 (0.27, 1.03) | 0.48 (0.38, 0.62) |
| **75-79** | 0.75 (0.37, 1.53) | 0.52 (0.28, 0.94) | 0.83 (0.46, 1.49) | 0.53 (0.35, 0.79) | 0.41 (0.25, 0.66) | 0.39 (0.11, 1.4) | 0.4 (0.25, 0.63) | 0.47 (0.23, 0.99) | 0.43 (0.33, 0.56) |
| **80-84** | 0.73 (0.34, 1.59) | 0.46 (0.24, 0.89) | 0.8 (0.42, 1.49) | 0.48 (0.31, 0.75) | 0.37 (0.22, 0.62) | 0.34 (0.09, 1.35) | 0.35 (0.21, 0.57) | 0.42 (0.19, 0.95) | 0.38 (0.28, 0.51) |
| **85-90** | 0.71 (0.31, 1.64) | 0.42 (0.21, 0.85) | 0.76 (0.39, 1.51) | 0.45 (0.28, 0.72) | 0.33 (0.19, 0.58) | 0.29 (0.07, 1.32) | 0.3 (0.17, 0.52) | 0.38 (0.16, 0.91) | 0.33 (0.24, 0.46) |
| **90-94** | 0.69 (0.28, 1.71) | 0.38 (0.18, 0.81) | 0.73 (0.35, 1.52) | 0.41 (0.25, 0.68) | 0.3 (0.16, 0.55) | 0.25 (0.05, 1.28) | 0.26 (0.15, 0.47) | 0.34 (0.13, 0.87) | 0.3 (0.21, 0.42) |
| **95 +** | 0.67 (0.25, 1.77) | 0.34 (0.15, 0.77) | 0.71 (0.32, 1.54) | 0.38 (0.22, 0.65) | 0.27 (0.14, 0.51) | 0.22 (0.04, 1.25) | 0.23 (0.12, 0.43) | 0.31 (0.11, 0.84) | 0.26 (0.18, 0.38) |
| **Period** |  |  |  |  |  |  |  |  |  |
| **1992-1996** | 1.03 (0.77, 1.37) | 1.25 (0.99, 1.59) | 1.08 (0.82, 1.41) | 1.01 (0.86, 1.19) | 1.12 (0.94, 1.35) | 1.46 (0.96, 2.22) | 1.23 (1.05, 1.43) | 1.1 (0.86, 1.4) | 1.26 (1.13, 1.39) |
| **1997-2001** | 0.94 (0.71, 1.24) | 1.11 (0.88, 1.4) | 1.03 (0.78, 1.35) | 1.01 (0.86, 1.18) | 1.03 (0.87, 1.23) | 1.16 (0.78, 1.72) | 1.1 (0.95, 1.27) | 1.06 (0.86, 1.32) | 1.14 (1.03, 1.26) |
| **2002-2006** | 1 (1, 1) | 1 (1, 1) | 1 (1, 1) | 1 (1, 1) | 1 (1, 1) | 1 (1, 1) | 1 (1, 1) | 1 (1, 1) | 1 (1, 1) |
| **2007-2011** | 0.97 (0.74, 1.28) | 0.87 (0.69, 1.11) | 0.96 (0.72, 1.28) | 1.04 (0.89, 1.22) | 0.96 (0.81, 1.14) | 0.88 (0.59, 1.32) | 0.92 (0.79, 1.06) | 0.92 (0.74, 1.14) | 0.85 (0.77, 0.95) |
| **2012-2016** | 0.9 (0.66, 1.21) | 0.79 (0.61, 1.02) | 0.91 (0.67, 1.22) | 0.77 (0.65, 0.92) | 0.74 (0.61, 0.9) | 0.77 (0.49, 1.21) | 0.69 (0.58, 0.82) | 0.71 (0.55, 0.92) | 0.77 (0.69, 0.86) |
| **2017-2021** | 0.87 (0.63, 1.21) | 0.76 (0.58, 1) | 0.88 (0.64, 1.21) | 0.67 (0.55, 0.81) | 0.66 (0.53, 0.82) | 0.7 (0.42, 1.19) | 0.63 (0.52, 0.76) | 0.67 (0.5, 0.89) | 0.71 (0.63, 0.8) |
| **Cohort** |  |  |  |  |  |  |  |  |  |
| **1897-1901** | 2.07 (0, 196266.68) | 6.96 (0.01, 9573.97) | 1.88 (0, 1876.29) | 4.15 (0.03, 515.65) | 7.72 (0.03, 1721.64) | 5.32 (0, 2563959300.72) | 6.38 (0.03, 1580.53) | 5.55 (0, 672422.99) | 6.6 (0.35, 125.41) |
| **1902-1906** | 1.88 (0.01, 367.51) | 3.95 (0.1, 154.12) | 1.55 (0.06, 41.56) | 3.46 (0.29, 41.4) | 5.97 (0.36, 99.29) | 5.27 (0, 57060.3) | 5.09 (0.31, 82.8) | 4.09 (0.01, 1201.63) | 4.75 (1.03, 22.02) |
| **1907-1911** | 1.71 (0.09, 32.71) | 2.92 (0.3, 28.64) | 1.39 (0.19, 10.25) | 2.97 (0.64, 13.71) | 4.61 (0.8, 26.58) | 4.81 (0.03, 692.57) | 4.35 (0.72, 26.41) | 3.3 (0.11, 96.57) | 3.84 (1.44, 10.21) |
| **1912-1916** | 1.58 (0.23, 10.66) | 2.46 (0.5, 12.13) | 1.33 (0.33, 5.29) | 2.64 (0.96, 7.3) | 3.7 (1.1, 12.43) | 4.21 (0.18, 96.52) | 3.77 (1.09, 13.06) | 2.79 (0.34, 22.71) | 3.38 (1.68, 6.81) |
| **1917-1921** | 1.36 (0.32, 5.84) | 2.15 (0.61, 7.53) | 1.22 (0.42, 3.52) | 2.24 (1.02, 4.91) | 3.07 (1.19, 7.89) | 3.67 (0.38, 35.58) | 3.21 (1.25, 8.25) | 2.42 (0.55, 10.59) | 2.98 (1.72, 5.15) |
| **1922-1926** | 1.3 (0.46, 3.66) | 2.05 (0.79, 5.32) | 1.12 (0.49, 2.57) | 1.96 (1.08, 3.54) | 2.67 (1.31, 5.45) | 3.18 (0.58, 17.37) | 2.74 (1.34, 5.59) | 2.14 (0.74, 6.15) | 2.72 (1.8, 4.13) |
| **1927-1931** | 1.21 (0.6, 2.44) | 1.94 (0.97, 3.89) | 1.05 (0.55, 1.98) | 1.71 (1.1, 2.66) | 2.26 (1.31, 3.9) | 2.73 (0.69, 10.79) | 2.4 (1.38, 4.16) | 1.9 (0.89, 4.06) | 2.4 (1.75, 3.29) |
| **1932-1936** | 1.16 (0.65, 2.08) | 1.76 (1.01, 3.06) | 1.03 (0.6, 1.78) | 1.51 (1.03, 2.21) | 1.95 (1.23, 3.09) | 2.3 (0.76, 6.92) | 2.12 (1.35, 3.33) | 1.7 (0.93, 3.12) | 2.08 (1.6, 2.71) |
| **1937-1941** | 1.12 (0.68, 1.87) | 1.53 (0.96, 2.45) | 1.03 (0.64, 1.66) | 1.36 (0.99, 1.87) | 1.67 (1.12, 2.48) | 1.86 (0.72, 4.79) | 1.81 (1.23, 2.67) | 1.52 (0.91, 2.53) | 1.78 (1.42, 2.24) |
| **1942-1946** | 1.09 (0.66, 1.8) | 1.34 (0.84, 2.15) | 1.03 (0.65, 1.63) | 1.21 (0.9, 1.63) | 1.44 (0.99, 2.1) | 1.57 (0.68, 3.62) | 1.53 (1.06, 2.21) | 1.36 (0.85, 2.16) | 1.48 (1.19, 1.83) |
| **1947-1951** | 1.04 (0.66, 1.66) | 1.2 (0.76, 1.9) | 1.01 (0.65, 1.58) | 1.15 (0.87, 1.52) | 1.28 (0.89, 1.82) | 1.33 (0.61, 2.9) | 1.31 (0.93, 1.85) | 1.22 (0.8, 1.88) | 1.33 (1.08, 1.64) |
| **1952-1956** | 1.02 (0.69, 1.52) | 1.09 (0.75, 1.6) | 1.01 (0.68, 1.5) | 1.06 (0.83, 1.35) | 1.12 (0.84, 1.51) | 1.16 (0.58, 2.34) | 1.14 (0.86, 1.53) | 1.1 (0.77, 1.58) | 1.15 (0.97, 1.37) |
| **1957-1961** | 1 (1, 1) | 1 (1, 1) | 1 (1, 1) | 1 (1, 1) | 1 (1, 1) | 1 (1, 1) | 1 (1, 1) | 1 (1, 1) | 1 (1, 1) |
| **1962-1966** | 1 (0.69, 1.44) | 0.93 (0.67, 1.28) | 0.99 (0.68, 1.46) | 0.94 (0.75, 1.18) | 0.93 (0.71, 1.2) | 0.91 (0.5, 1.65) | 0.89 (0.7, 1.14) | 0.91 (0.67, 1.25) | 0.9 (0.77, 1.06) |
| **1967-1971** | 1 (0.67, 1.51) | 0.86 (0.61, 1.22) | 1 (0.66, 1.51) | 0.9 (0.71, 1.15) | 0.87 (0.66, 1.14) | 0.84 (0.45, 1.54) | 0.8 (0.62, 1.03) | 0.84 (0.61, 1.17) | 0.84 (0.71, 0.98) |
| **1972-1976** | 0.99 (0.64, 1.54) | 0.81 (0.56, 1.17) | 0.97 (0.62, 1.52) | 0.88 (0.68, 1.13) | 0.83 (0.63, 1.11) | 0.77 (0.41, 1.45) | 0.72 (0.55, 0.94) | 0.78 (0.55, 1.1) | 0.78 (0.66, 0.93) |
| **1977-1981** | 0.97 (0.61, 1.54) | 0.73 (0.49, 1.08) | 0.92 (0.57, 1.5) | 0.84 (0.65, 1.1) | 0.81 (0.6, 1.09) | 0.7 (0.36, 1.35) | 0.64 (0.49, 0.85) | 0.72 (0.5, 1.03) | 0.72 (0.6, 0.86) |
| **1982-1986** | 0.98 (0.61, 1.58) | 0.65 (0.43, 0.98) | 0.88 (0.52, 1.48) | 0.81 (0.61, 1.07) | 0.79 (0.58, 1.08) | 0.63 (0.31, 1.28) | 0.58 (0.44, 0.77) | 0.66 (0.45, 0.97) | 0.65 (0.54, 0.79) |
| **1987-1991** | 1 (0.6, 1.66) | 0.6 (0.39, 0.91) | 0.84 (0.47, 1.48) | 0.79 (0.58, 1.06) | 0.8 (0.58, 1.11) | 0.59 (0.28, 1.24) | 0.53 (0.4, 0.71) | 0.61 (0.41, 0.9) | 0.61 (0.5, 0.74) |
| **1992-1996** | 1.03 (0.59, 1.79) | 0.59 (0.38, 0.93) | 0.8 (0.42, 1.51) | 0.78 (0.57, 1.09) | 0.82 (0.58, 1.15) | 0.58 (0.27, 1.26) | 0.52 (0.38, 0.7) | 0.56 (0.37, 0.86) | 0.59 (0.48, 0.73) |
| **1997-2001** | 1.01 (0.53, 1.95) | 0.54 (0.33, 0.89) | 0.73 (0.34, 1.55) | 0.74 (0.51, 1.07) | 0.75 (0.52, 1.09) | 0.46 (0.2, 1.05) | 0.41 (0.3, 0.56) | 0.49 (0.31, 0.78) | 0.51 (0.41, 0.64) |
| **2002-2006** | 0.98 (0.44, 2.17) | 0.44 (0.24, 0.78) | 0.65 (0.26, 1.64) | 0.62 (0.4, 0.97) | 0.59 (0.38, 0.91) | 0.36 (0.15, 0.9) | 0.29 (0.21, 0.41) | 0.41 (0.24, 0.7) | 0.43 (0.34, 0.54) |
| **2007-2011** | 0.88 (0.32, 2.45) | 0.31 (0.16, 0.62) | 0.52 (0.16, 1.76) | 0.47 (0.28, 0.8) | 0.4 (0.24, 0.66) | 0.23 (0.08, 0.64) | 0.21 (0.14, 0.3) | 0.32 (0.17, 0.62) | 0.32 (0.25, 0.42) |
| **2012-2016** | 0.64 (0.12, 3.26) | 0.21 (0.09, 0.49) | 0.33 (0.05, 2.18) | 0.31 (0.15, 0.65) | 0.27 (0.15, 0.52) | 0.1 (0.03, 0.35) | 0.17 (0.11, 0.25) | 0.24 (0.09, 0.61) | 0.21 (0.15, 0.28) |
| **2017-2021** | 0.31 (0.01, 12.5) | 0.15 (0.04, 0.57) | 0.11 (0, 8.54) | 0.17 (0.04, 0.7) | 0.16 (0.06, 0.44) | 0.03 (0, 0.24) | 0.11 (0.07, 0.18) | 0.11 (0.02, 0.85) | 0.12 (0.08, 0.18) |
